# Genetic profiling of patients with atopic dermatitis reveals immune and skin barrier variants associated with generalized eczema and optimal response to Dupilumab therapy

**DOI:** 10.3389/fimmu.2026.1788831

**Published:** 2026-04-10

**Authors:** Martina Morelli, Giovanni Luca Scaglione, Anna Dattolo, Marco Galluzzo, Claudia Scarponi, Gaia Moretta, Alessia Provini, Filomena Russo, Barbara Cocuroccia, Donatella Sordi, Marina Talamonti, Valeria Villella, Fabio Romano Selvi, Laura Mercurio, Claudia Paganini, Edoardo Mortato, Luca Bianchi, Ornella De Pità, Sabatino Pallotta, Enrico Scala, Cristina Albanesi, Stefania Madonna

**Affiliations:** 1 Laboratory of Experimental Immunology Unit, IDI-IRCCS, Rome, Italy; 2 Department of Systems Medicine, University of Rome “Tor Vergata”, Rome, Italy; 3 Dermatology Unit, Fondazione Policlinico “Tor Vergata”, Rome, Italy; 4 Department of Dermatology Unit, Istituto Dermopatico dell’Immacolata - Istituto di Ricovero e Cura a Carattere Scientifico (IDI-IRCCS), Rome, Italy; 5 Molecular Allergology Unit, IDI-IRCCS, Rome, Italy

**Keywords:** atopic dermatitis, dupilumab, filaggrin, generalized eczema, KIF3A, pharmacogenomics, single-nucleotide polymorphisms

## Abstract

**Background:**

Atopic dermatitis (AD) is a clinically heterogeneous immune-mediated skin disorder. The lack of reliable clinical stratification tools and predictive biomarkers for therapeutic response remains a critical unmet need to optimize treatment and advance precision dermatology.

**Objective:**

To investigate the association between genetic variants and AD phenotypes and response to Dupilumab as a potential approach to individualize therapy in moderate-to-severe AD.

**Methods:**

120 patients with moderate-to-severe AD treated with Dupilumab were enrolled at two Italian centers. Clinical data, including phenotypes, severity, and comorbidities, were collected at baseline and after 48 weeks treatment with Dupilumab. Its efficacy was assessed as EASI75 and EASI90 achievements at week 48. Genotyping of 521 SNPs was performed using next-generation sequencing (NGS). Cluster analysis and univariate logistic regression explored associations between genetic profiles and clinical variables.

**Results:**

NGS-based genotyping enabled the stratification of patients into four distinct genetic clusters, each characterized by unique combinations of polymorphisms in skin barrier–related genes, namely *FLG* and *KIF3A*. Specific SNPs in additional barrier-related genes (RPTN rs3001978, TH2LCRR rs2158177 and rs3091307) and Th2 pathway genes (IL4R rs1805016, STAT6 rs324011) were differentially distributed across the clusters. A combined variant pattern in *KIF3A* and *FLG* was strongly associated with a generalized eczema phenotype. Some allelic variants of genes were found to be associated with conjunctivitis (*FLG*), rhinitis (*KIF3A*), bronchial asthma (*IL5RA*) comorbidities and high IgE levels (*ADAM33*). Notably, FLG rs749682384 and IL6R rs2228145 variants emerged as predictors of favorable response to Dupilumab, while RPTN rs3001978 and TSLP rs2289276 variants were associated with a therapy failure.

**Conclusions:**

This study identified genetic signatures in skin barrier and Th2 pathway genes associated with clinical phenotypes and differential responses to Dupilumab in AD. Findings support incorporating genotypic profiling into practice to guide personalized therapy in moderate-to-severe AD.

## 1 Introduction

Atopic dermatitis (AD) is a chronic, relapsing immune-mediated skin disorder that affects up to 20% of children and approximately 10% of adults worldwide. Clinically, it is characterized by intense pruritus, xerosis, and eczematous lesions with variable distribution patterns, including flexural, generalized, head/neck, or hand involvement. The extrinsic form of AD, distinguished from its intrinsic counterpart, is marked by elevated total and specific IgE levels to environmental and food allergens, and frequently coexists with other atopic conditions such as asthma, allergic rhinitis, and allergic conjunctivitis (1–3).

The pathogenesis of AD is multifactorial, involving a complex interplay between environmental exposures, epidermal barrier dysfunction, and dysregulated immune responses, particularly type-2 inflammation (4). Alongside T helper (Th)2 responses, additional immune axes including Th1, Th17, and Th22 pathways contribute to the heterogeneity of AD phenotypes, reflecting its broad clinical and immunological spectrum.

A substantial body of genetic evidence has underscored a strong hereditary component in AD susceptibility. Genome-wide association studies (GWAS) have identified over 30 risk loci, many of which map to genes involved in skin barrier function (e.g., *FLG, KIF3A, HRNR-RPTN*) *(*5), immune signaling (e.g., *IL4, IL13*), cytokine receptors (e.g., *IL6R, IL5RA*), and epithelial-derived alarmins (e.g., *TSLP*) *(*6). Subsequent GWAS have confirmed these findings (7, 8) and expanded the list of susceptibility variants to a total of 81, with 29 novel single-nucleotide polymorphisms (SNPs) recently linked to Th2-dominant immune pathways (9, 10). Despite these advances, the clinical implementation of genetic data for disease stratification and personalized therapy in AD remains limited.

Over the past decade, the therapeutic landscape of AD has evolved significantly with the introduction of targeted biologics such as IL-4/IL-13 inhibitors and Janus kinase inhibitors, offering new treatment avenues for patients with moderate-to-severe disease. However, real-life clinical response to these agents remains variable: approximately one-third of patients fail to achieve optimal skin clearance, often necessitating therapeutic switching and incurring increased healthcare burden (11, 12).

While genetic research has elucidated key molecular pathways involved in AD pathophysiology, the translation of these insights into predictive biomarkers for treatment response is still at an early stage. In particular, the potential of germline variants to serve as pharmacogenetic markers for response to IL-4/IL-13-targeting biologics has yet to be fully explored, representing a promising frontier in precision dermatology.

To address this gap, we conducted a candidate-gene study to investigate the association between 521 SNPs in genes implicated in immune regulation, epithelial signaling, and skin barrier function, and both clinical characteristics and long-term response to Dupilumab therapy. This targeted genotyping panel was applied to a cohort of 120 adult patients with moderate-to-severe AD receiving Dupilumab, with the aim of identifying genotype–phenotype correlations and potential predictive biomarkers of therapeutic efficacy.

## 2 Methods

### 2.1 Patients

This multicenter, cross-sectional study was conducted at the Dermatology Units of IDI-IRCCS Hospital and Fondazione Policlinico Tor Vergata (PTV) in Rome, Italy. The study received ethical approval by Territorial Ethics Committee Lazio Area 5 of Rome (Protocol No. 09/CE/2023), and written informed consent was obtained from all participants. A total of 120 adult patients with moderate-to-severe AD eligible for Dupilumab therapy were prospectively enrolled. All patients were of Italian ancestry, as confirmed by genealogical information reported for both parents and grandparents. In addition, the genetic homogeneity of our cohort was also confirmed by evaluating ancestry consistency by comparing the allele frequencies of the SNPs in AD population (variant allele frequency, VAF) with the available minor allele frequencies (MAF) reported in the 1000 Genomes Project across different populations (European, African, East Asian, and Admixed American) (data not shown). Therapeutic regimen of Dupilumab was according to standard clinical practice (initial 600 mg dose followed by 300 mg every two weeks). For each patient, personal data, as well as anthropometric and clinical data were collected and annotated. The severity of AD and response to Dupilumab were evaluated using the Eczema Area and Severity Index (EASI) score at baseline and after 48 weeks. Clinical efficacy was assessed in terms of the 75% and 90% improvement of EASI score compared to baseline (EASI75 and EASI90). Saliva samples were collected using the Oragene^®^ DNA self-collection kit (DNA Genotek Inc., Canada) to isolate DNA.

### 2.2 Sample processing and genotyping

Genomic DNA was extracted using the QIAamp DNA extraction kit (QIAGEN, Germany), and 10 ng of DNA were subjected to next-generation sequencing (NGS). The customized panel comprised 122 SNPs distributed across 94 amplicons (average size ~275 bp) corresponding to genes involved in immune regulation, inflammatory pathways, and skin barrier function. Sequencing of amplicons allowed the identification of an additional 399 SNPs located near the primary SNPs. The targeted panel was intentionally enriched for SNPs within *FLG* and *KIF3A*, given their established roles as major determinants of skin barrier function and AD risk. The SNP array analysis therefore identified a total of 521 genetic variants.

SNP panel was designed based on a comprehensive review of genome-wide association studies (GWAS) and previous reports describing associations between AD and specific SNPs (6, 7, 13, 14). Relevant variants were those showing the strongest association with AD in populations of European ancestry (p < 5×10^-8^) and were further confirmed using the ClinVar and DisGeNET databases. This preselection of SNPs classifies the study as a candidate SNP study, which is inherently limited by prior knowledge.

Sequencing was performed across multiple runs on the Ion GeneStudio™ S5 Plus platform (Thermo Fisher Scientific, USA) (15). Libraries were amplified by the Ion AmpliSeq™ Library kit Plus (Thermo Fisher) and quantified using the Qubit 4 Fluorometer and 2100 Bioanalyzer with dsDNA HS assay and High Sensitivity DNA kit (Thermo Fisher), respectively. Sequencing data were processed with Ion Torrent Suite software v.5.10.

Although no formal inter-batch correction was applied, uniform quality control thresholds (read depth > 30X and call rate > 95%) were applied to the aggregated dataset. A total of 367 high-quality SNPs was used for statistical analyses (**Supplementary Table 1**).

Given that all samples were processed using the same targeted panel, library preparation protocol, and sequencing platform in a single laboratory, systematic batch effects are unlikely to have substantially influenced the results.

All SNPs were annotated according to their genomic positions using the human reference genome assembly hg19 (GRCh37).

### 2.3 Bioinformatics and statistics

Sample size estimation for the comparison between carriers and non-carriers of previously validated AD-associated SNPs was conducted using G*Power (version 3.1.9.7, Universität Düsseldorf, Germany). Owing to the lack of published data on the associations between the polymorphisms and treatment response in AD patients, the power calculation was performed using data derived from a small subset of patients (n= 57) initially enrolled for our study. Based on this internal cohort data, the proportion of patients achieving EASI90 at week 48 was in line with a previous real-life study (66.8% of responders) (11), and among them the 38% were non-carriers and 68% carriers of a known AD-risk SNP rs2299007 (VAF:0.25 and MAF:0.23) (5). Under the assumptions of a two-sided α = 0.05, a statistical power of 80%, and a carrier-to-non-carrier ratio of 1.8, the required sample size was estimated to be n= 108 patients in total. After adjustment for an anticipated 10% dropout rate, the final target sample size was set at approximately 120 participants. This sample size was considered sufficient to ensure adequate statistical power for subsequent pharmacogenomic association analyses.

Heatmap analyses were performed to evaluate the distribution and clustering of SNPs across the patient cohort. Data matrices were normalized prior to hierarchical clustering using appropriate distance metrics and linkage methods. SNP genotypes were encoded as binary variables (0 = absence of the minor allele, 1 = presence of the minor allele in either heterozygous or homozygous state), thus corresponding to a dominant genetic model. In addition, SNPs were categorized according to their variant allele frequency (VAF), with variants showing VAF <10% defined as low-frequency SNPs and those with VAF ≥10% defined as high-frequency SNPs. Rows (SNPs) were hierarchically clustered using Euclidean distance with complete linkage, as implemented in the ComplexHeatmap package. Columns (patients) are ordered by cluster assignment (A–D) and were not subjected to hierarchical clustering. Visualizations were generated with the ComplexHeatmap and ggplot2 packages in R (version 4.3.3).

To explore multivariate genetic patterns among AD patients, Constrained Analysis of Principal Coordinates (CAP) was conducted based on Bray-Curtis dissimilarity matrices. Statistical significance was assessed through permutation testing, and ordinations were visualized as biplots emphasizing key SNP feature vectors contributing to group separation.

Principal Component Analysis (PCA) was applied to explore SNP distribution in AD population and to evaluate associations between SNP patterns and clinical phenotypes. After normalization, centering, and scaling of genotype data, variance structures were examined with confidence ellipses illustrating group dispersion in PCA space. All analyses were conducted using R.

Associations between SNPs and clinical variables, including response to Dupilumab were tested using univariate logistic regression analysis. All variables were adjusted for age, sex, smoking status, and allergen exposure, using SPSS v29 software (IBM SPSS, Chicago, IL). For each clinical variable, *p*-values were corrected for multiple testing using the Benjamini–Hochberg false discovery rate (FDR) procedure. Odds ratios (OR) with 95% confidence intervals (CI) were reported for all SNPs and clinical parameters. Quality control steps excluded SNPs and individuals with >10% missing genotype data. A p-value threshold of <0.05 was used to denote statistical significance.

## 3 Results

### 3.1 Bronchial asthma correlates with chronicity and conjunctivitis in AD, while high baseline EASI predicts better Dupilumab response

Overall, 120 adult patients of Italian ancestry with moderate-to-severe AD undergoing Dupilumab therapy were enrolled (**Table 1**). The mean age was 38.2 years, with an average disease duration of 20.6 years. Intrinsic and extrinsic AD phenotypes were equally represented. The prevalence of allergic comorbidities was high, including bronchial asthma in 35.0% of patients, allergic rhinitis in 52.5%, and allergic conjunctivitis in 40.8%. Generalized eczema was present in 31.7% of the cohort. At baseline, the mean EASI score was 31.0, with numerical rating scale (NRS) scores of 8.7 for pruritus and 7.1 for sleep disturbance. After 48 weeks of Dupilumab therapy, clinical outcomes significantly improved: 71.0% of patients achieved EASI75 and 57.0% reached EASI90.

**Table 1:** Demographic data and clinical characteristics of AD patients (*n* = 120).

Characteristics at baseline	
Male/Female (*n*)	58/62
	*Mean ± DS (range)*
Age (years)	38.2 ± 17.5 (11–82)
Age at disease onset (years)	17.4 ± 21.2 (0-80)
Duration of disease (years)	20.6 ± 13.9 (1-73)
BMI (*kg/m^2^*)	23.8 ± 4.0 (16-39.8)
Environmental exposures	*n (%)*
Tobacco Smoke	54 (45)
Allergen (≥1)	70 (58.3)
* Airborne	45 (37.5)
* Food	25 (20.8)
* Skin	19 (15.8)
IgE (T_0_)	*n (%)*
<200 (*UI/ml*)	45 (44.6)
>200 (*UI/ml*)	56 (55.4)
Comorbidities (T_0_)	*n (%)*
Bronchial Asthma	42 (35.0)
Rhinitis	63 (52.5)
Conjunctivitis	49 (40.8)
Clinical Phenotype (T_0_)	*n (%)*
Flexural regions dermatitis	24 (20.0)
Head and Neck dermatitis	12 (10.0)
Portrait dermatitis	4 (3.3)
Hand dermatitis	11 (9.2)
Generalized Eczema	38 (31.7)
Prurigo	16 (13.3)
Nummular Eczema	5 (4.2)
Erythroderma	5 (4.2)
Psoriasiform dermatitis	3 (2.5)
Lichen	1 (0.8)
EASI (T_0_)	31.0 ± 11.1 (15-72)
NRS Itching (T_0_)	8.7 ± 1.7 (0-10)
NRS Sleep (T_0_)	7.1 ± 3.2 (0-10)
Characteristics at 48 weeks of dupilumab treatment	
	*Mean ± DS (range)*
EASI (W48)	5.8 ± 7.9 (0-30)
NRS Itching (W48)	3.1 ± 3.0 (0-10)
NRS Sleep (W48)	1.8 ± 2.9 (0-10)
Treatment Response Criteria (W48)	*n (%)*
EASI75	76 (71.0)
EASI90	61 (57.0)

Univariable logistic regression analysis revealed several clinically meaningful associations (**Supplementary Table 2**). At baseline, allergic rhinitis was significantly associated with longer disease duration and the presence of bronchial asthma. In turn, bronchial asthma was strongly associated with conjunctivitis, reflecting a shared atopic endotype. Elevated sleep disturbance scores (NRS > 6) were significantly associated with high total IgE levels (≥200 U/mL), suggesting a link between systemic atopy and quality-of-life impairment.

Importantly, baseline disease severity emerged as a predictor of treatment response. Patients with EASI scores ≥ 24 at baseline had significantly higher odds of achieving EASI90 at week 48 of Dupilumab treatment (OR 6.21, 95% CI 1.25-30.83; *p* = 0.013; **Supplementary Table 2**). This observation reinforces the hypothesis that individuals with higher baseline inflammatory burden may derive greater benefit from IL-4/IL-13-targeted therapy.

### 3.2 Genetic patterns in *FLG* and *KIF3A* delineate four molecular clusters in AD patients

**Figure 1:**
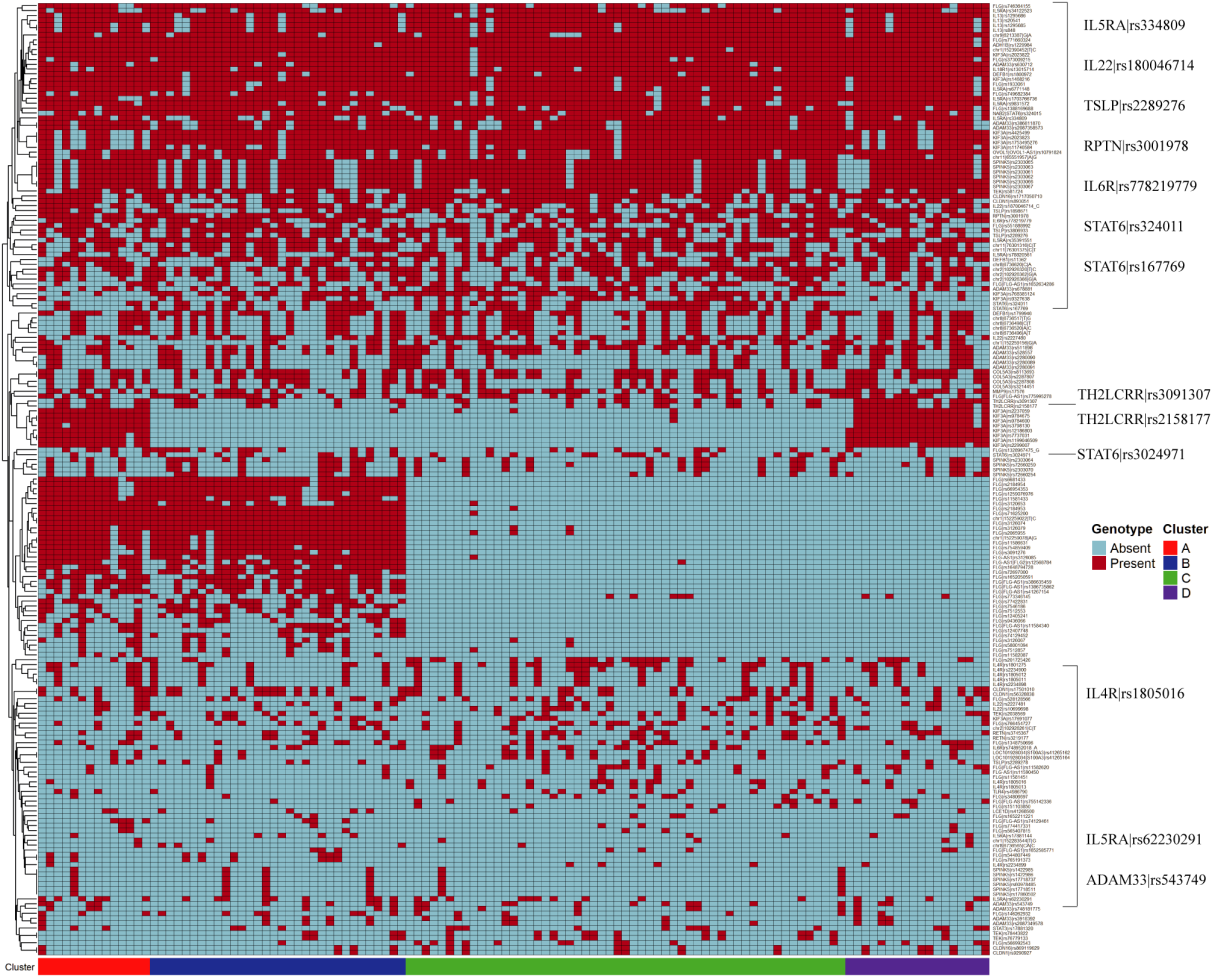
Heatmap displaying the distribution of 195 SNPs present in over 5% of the AD patient cohort. Each column corresponds to an individual patient, and each row to a specific SNP locus. Genotypes are color-coded to reflect the presence or absence of the minor allele. Patients are grouped according to their cluster assignment based on shared SNP patterns, as indicated by the colored annotation bar below the heatmap. Rows (SNPs) were hierarchically clustered using Euclidean distance with complete linkage. Columns (patients) are ordered by cluster assignment (A–D) and were not subjected to hierarchical clustering.

Heatmap analysis of SNPs identified 195 out of 367 allelic variants, present in more than 5% of the study cohort, among which 130 were highly frequent, occurring in approximately 75% of patients (**Figure 1**; **Supplementary Table 1**). Unsupervised clustering of patients based on SNP distribution revealed four distinct genetic clusters, primarily driven by variants in the FLG and KIF3A genes, two key regulators of skin barrier function. These variants were either broadly distributed across the cohort or selectively enriched in specific patient subsets. Notably, 37 SNPs in *FLG* and *KIF3A* exhibited high discriminatory power, allowing the definition of four genetic endotypes characterized by distinct combinations of KIF3A and FLG genotypes (**Supplementary Figure 1**, **Supplementary Table 3**). Cluster A was defined by the co-occurrence of KIF3A and FLG variants (KIF3A^+^/FLG^+^), cluster B by FLG variants only (KIF3A^-^/FLG^+^), and cluster D by KIF3A variants only (KIF3A^+^/FLG^-^). Cluster C lacked relevant SNPs in both genes (KIF3A^-^/FLG^-^).

Additional high-frequency SNPs involved skin barrier genes (RPTN rs3001878) and immune-related loci including *IL5RA* (rs334809), *IL22* (rs180046714), *TSLP* (rs2289276), *IL6R* (rs2228145), and *STAT6* (rs324011, rs167768) (**Figure 1**; **Supplementary Table 1**). Lower-frequency variants included SNPs in *TH2LCRR* (rs3091307, rs2158177), *IL4R* (rs1805016), *ADAM33* (rs543749), and additional loci within *STAT6* (rs3024971) and *IL5RA* (rs62230291).

**Figure 2:**
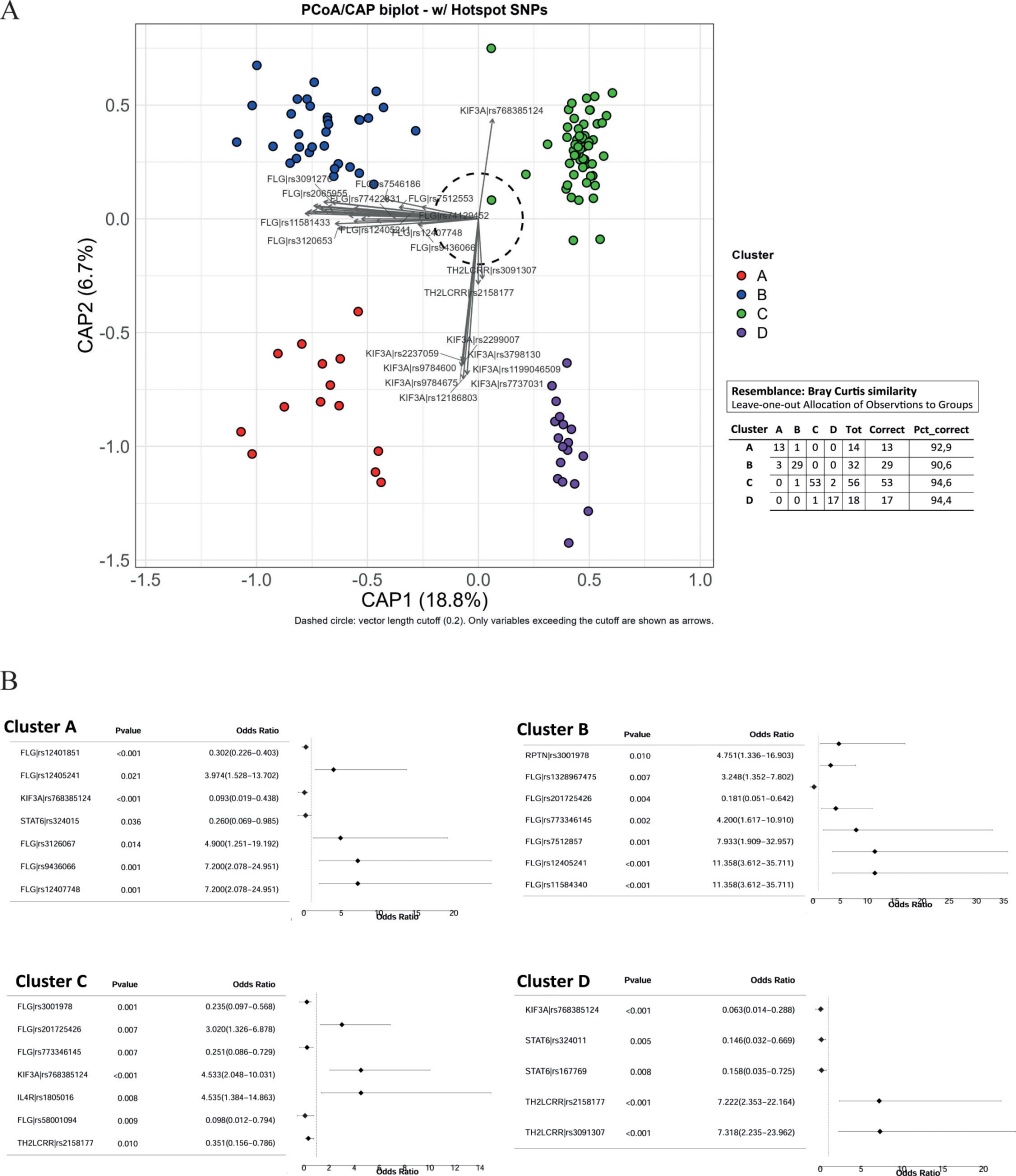
Constrained analysis of principal coordinates (CAP) performed on the genotype matrix of 367 SNPs across 120 patients. Each point represents a single patient projected in the constrained ordination space using Bray-Curtis dissimilarity, with colors indicating genetic clusters. Feature vectors (arrows) represent SNP hotspots with a correlation exceeding 0.2 with the canonical axes, identifying key variants driving cluster separation **(A)**. Forest plots showing the association between selected SNPs and the four immune clusters (Clusters A–D). For each SNP, the odds ratio (OR) and 95% confidence interval (CI) were calculated using logistic regression analysis. Points represent the estimated OR and horizontal lines indicate the 95% CI. *p* values < 0.05 were considered significant **(B)**.

To further explore the genetic structure of the cohort, we performed principal coordinate analysis (CAP) based on the 195 SNPs. This confirmed the segregation of patients into four spatially distinct clusters along the first two axes (**Figure 2A**). Cluster A (KIF3A^+^/FLG^+^) was localized predominantly in the lower-left quadrant (92.9%), cluster B (KIF3A^-^/FLG^+^) in the upper-left quadrant (90.6%), and cluster D (KIF3A^+^/FLG^-^) in the lower-right quadrant. Cluster C (KIF3A^-^/FLG^-^), lacking variants in both genes, was positioned in the upper-right quadrant. Overlapping distributions of shared SNPs between clusters A and B (FLG variants), and clusters A and D (KIF3A variants), further highlighted genetic continuity among subsets. Specifically, all FLG and KIF3A variants were detected in the heatmap analysis, except for five specific SNPs (rs9436066, rs74129452, rs12407748, rs12405241, rs768385124). Among them, some were confirmed by univariate logistic regression (**Figure 2B**). For instance, rs9436066 and rs12405241 in FLG were significantly associated with clusters A and B, respectively. A pathogenic variant in *KIF3A* (rs768385124) strongly characterized cluster C, while rs2158177 and rs3091307 SNPs in *TH2LCRR* were markers of cluster D. Further associations were found for other SNPs in *FLG* and KIF3A, as well as for rs324015 in *STAT6*, rs3001978 in *RPTN*, and rs1805016 in *IL4R* (**Figure 2B**). In contrast, the STAT6 protective variants rs324011 and rs167769 were exclusively represented in cluster D.

**Figure 3:**
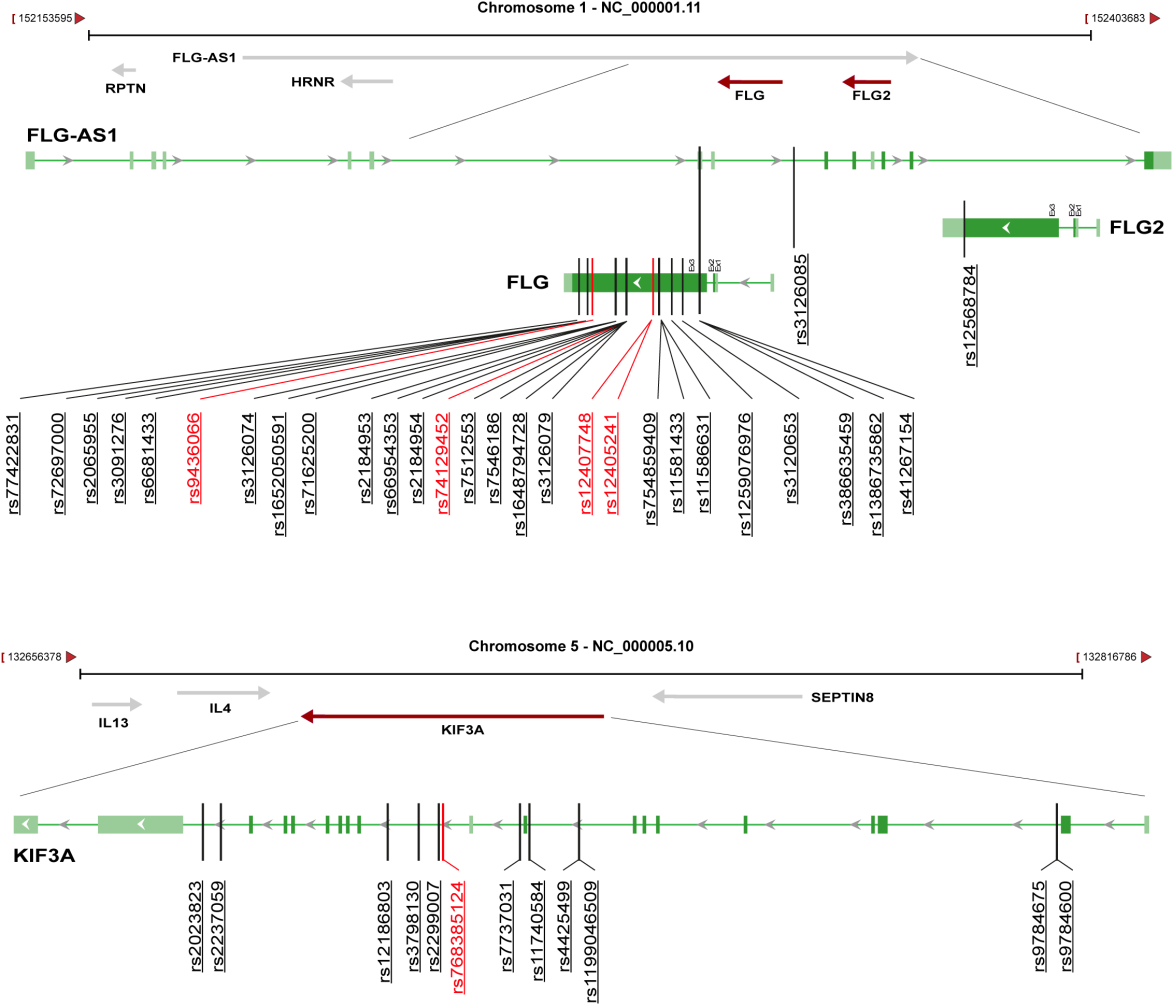
Genomic location of SNPs within the FLG and KIF3A loci across clusters A–D. A schematic representation of all identified SNPs found associated with the four AD patient clusters, and their relative genomic positions (vertical lines) within *FLG/FLG2/FLG-AS1* and *KIF3A* on chromosomes 1 and 5, respectively, are shown. Genes are shown with exons (green boxes) and introns (connecting green lines), with arrows indicating transcriptional orientation. Variants highlighted in red emerged as significant in both CAP and univariate analysis.

The integration of heatmap-based clustering, genetic association analyses and genomic localization highlighted SNPs in two major susceptibility FLG/FLG-AS1 and KIF3A loci, which differently distributed among the four clusters. In particular, genetic variants within the FLG locus were detected in patients from both clusters A and B and were predominantly located in exon 3 of FLG and FLG2, as well as in the overlapping antisense sequence FLG-AS1 (**Figure 3**). In contrast, KIF3A-associated variants, mainly distributed across clusters C and D, and were located in intronic and untranslated regions (**Figure 3**).

These findings support the existence of genetically distinct molecular endotypes within AD, driven by differential combinations of barrier and immune-related gene variants, which may underlie interindividual variability in disease phenotype and treatment response.

### 3.3 *KIF3A* and *FLG* variants are associated with the generalized eczema phenotype in AD

**Figure 4:**
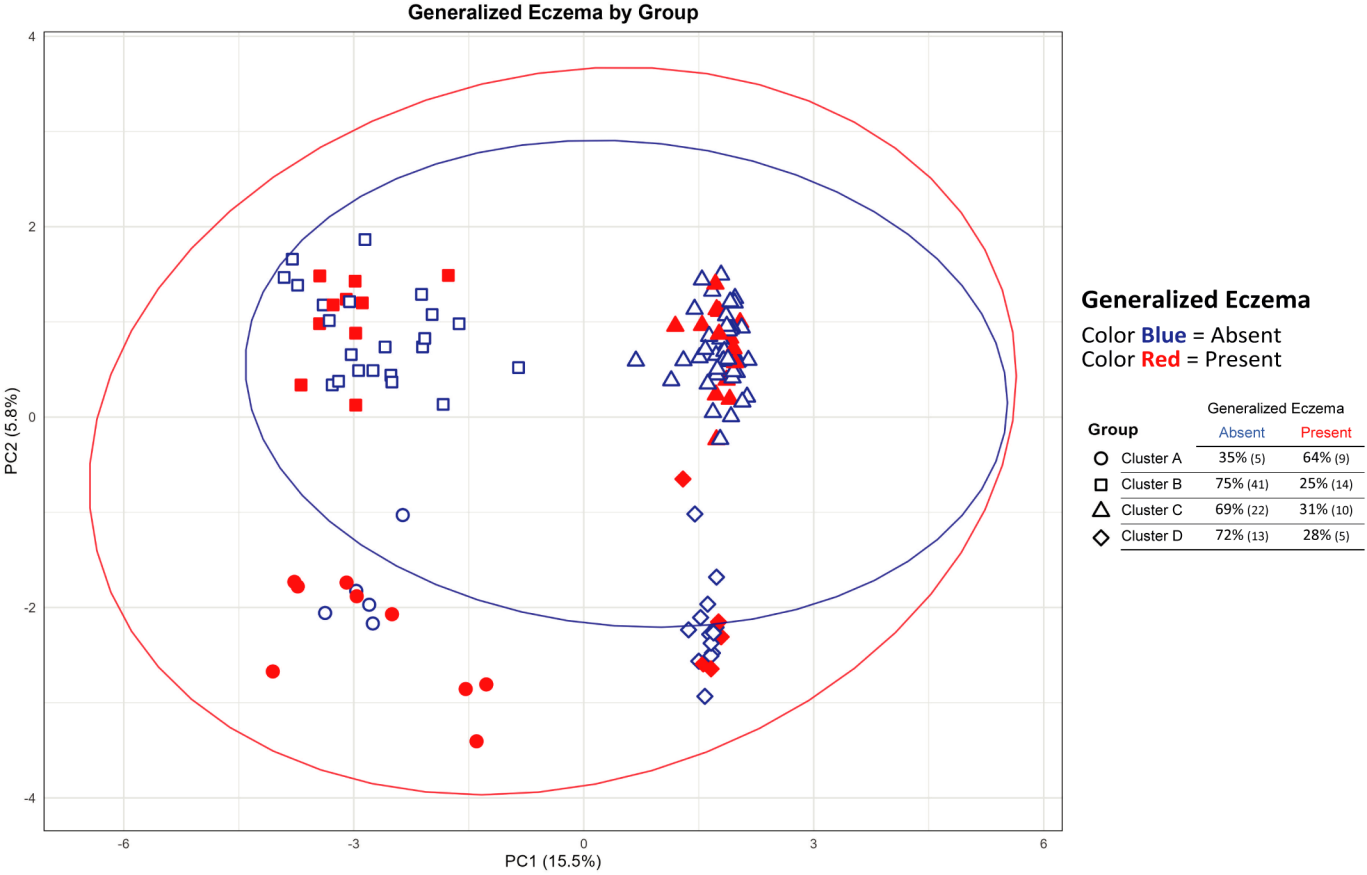
Principal component analysis (PCA) of patients based on 367 SNPs, revealing four genetic clusters influenced predominantly by 37 hotspot SNPs within *FLG* and *KIF3A*. Clusters are positioned as follows: cluster A (lower left), cluster B (upper left), cluster C (upper right), and cluster D (lower right). Red and blue symbols denote presence or absence of generalized eczema, respectively.

To explore potential associations between genetic stratification and clinical phenotypes, PCA was performed on the entire set of 367 SNPs. This analysis confirmed the presence of four genetically distinct clusters, driven predominantly by 37 hotspot variants in FLG and KIF3A (**Figure 4**; **Supplementary Table 3**). As observed in previous analyses, cluster A (KIF3A^+^/FLG^+^) located in the lower left quadrant, cluster B in the upper left, cluster C in the upper right, and cluster D in the lower right of the PCA plot.

Notably, the prevalence of generalized eczema differed significantly across clusters. Cluster A showed the highest proportion of affected individuals, with 64% of patients exhibiting a generalized distribution of eczema. In contrast, clusters B, C, and D were predominantly composed of patients without generalized involvement, with 69–75% of patients in these groups lacking this phenotype. The PCA pattern distribution in the four clusters was completely lost when SNPs in *FLG* and *KIF3A* were removed from analysis (data not shown).

The association between cluster A and the generalized eczema phenotype was further supported by univariate logistic regression, yielding an odds ratio of 4.717. No other clinical variables showed clear segregation across clusters in the PCA space, suggesting a specific link between the KIF3A/FLG genetic pattern and the extent of skin involvement.

### 3.4 Genetic variants in *FLG*, *RPTN, TSLP*, and *IL6R* are associated with favorable response to Dupilumab

Univariate logistic regression analysis revealed several SNPs significantly associated with baseline clinical phenotypes (**Table 2**). Generalized eczema exhibited strong associations with pathogenic FLG variants (rs1328967475, rs3120653, rs2011331) and with a protective KIF3A variant (rs11740584), both of which were also highlighted in the CAP-based clustering analysis (**Figure 2**, **3**). Additional associations were identified with *IL5RA* (rs334809) and *IL22* (rs1870046714), two variants highly enriched in our AD cohort (allele frequency ∼0.75) but rare in the general population (MAF <0.5) (**Supplementary Figure 2**), suggesting possible population-specific risk factors.

**Table 2:** Logistic regression analysis of clinical variables and treatment response after 48 weeks of Dupilumab.

Characteristic	Gene dbSNP	ORs	[95% CI]	*p*-value	FDR
Bronchial Asthma	IL5RA|rs62230291	2.613	[1.137-6.584]	0.042	0.680
Conjunctivitis	FLG-AS1|rs11584340	3.627	[1.124-11.70]	0.031	0.609
	FLG-AS1|rs2011331	3.701	[1.434-9.549]	0.007	0.190
	FLG-AS1|rs11588170	3.320	[1.268-8.695]	0.015	0.370
Rhinitis	FLG-AS1|rs1652634286	2.118	[1.101-4.806]	0.050	0.684
	FLG|rs566992543	0.198	[0.055-0.718]	0.014	0.350
Generalized Eczema	FLG|rs1328967475	4.564	[1.824-11.42]	0.001	0.029
	FLG|rs3120653	2.655	[1.140-6.182]	0.024	0.524
	FLG-AS1|rs2011331	3.032	[1.242-7.403]	0.015	0.370
	KIF3A|rs11740584	0.404	[0.155-0.952]	0.045	0.684
	IL5RA|rs334809	0.304	[0.107-0.861]	0.025	0.538
	IL22|rs1870046714	0.332	[0.144-0.765]	0.010	0.262
IgE levels	FLG|rs766454727	3.340	[1.045-11.225]	0.050	0.684
	ADAM33|rs543749	3.659	[1.057-12.67]	0.041	0.677
EASI75 w48	FLG|rs749682384	8.705	[1.951-38.84]	0.005	0.139
	irFLG|rs11204972	2.862	[1.118-8.048]	0.046	0.687
	RPTN|rs3001978	0.313	[0.093-0.950]	0.040	0.673
	TSLP|rs2289276	0.372	[0.143-0.967]	0.043	0.683
EASI90 w48	FLG|rs749682384	3.582	[1.042-15.234]	0.044	0.851
	irFLG|rs11204972	2.617	[1.173-6.382]	0.034	0.636
	FLG|rs528128566	0.298	[0.115-0.767]	0.012	0.307
	IL6R|rs2228145	4.122	[1.702-9.980]	0.002	0.058

Regarding comorbidities, bronchial asthma was significantly associated with the causative IL5RA variant rs62230291 (OR = 3.400, p = 0.007). Conjunctivitis occurred more frequently in patients carrying FLG-AS1 variants (rs11584340, rs2011331, rs11588170; OR range: 2.855–3.514), while allergic rhinitis was linked to FLG-AS1 rs1652634286 (OR = 2.313, p = 0.029). Conversely, protective effects were associated with FLG rs566992543 and KIF3A rs1468216, the latter found in approximately 90% of AD patients versus less than 25% in the European general population (**Supplementary Figure 2**). Elevated total IgE levels (>200 IU/mL) were associated with FLG rs766454727 and ADAM33 rs543749 (OR = 2.788 and OR = 3.789, respectively) (**Table 2**).

Importantly, SNPs within *FLG, RPTN, TSLP*, and *IL6R* genes were significantly associated with therapeutic outcomes following Dupilumab treatment at week 48, based on EASI75 and EASI90 achievement. Among the FLG variants, including rs11204972, rs528128566 and rs749682384, this last exhibited the strongest predictive value, with an OR of 8.464 for achieving EASI75 and 4.070 for EASI90 (**Table 2**). Also these variants were located at the 3’end of exon 3 of FLG. Likewise, rs2228145 in *IL6R* was significantly associated with EASI90 response. In contrast, rs3001978 in *RPTN* and rs2289276 in *TSLP* were linked to reduced probability of achieving EASI75. It is important to note that many associations with specific SNPs showed reduced statistical significance after FDR correction (**Table 2**). In addition, both rs2228145 and rs3001978 were enriched in the study AD population (allele frequency ∼70%) relative to the general population (∼40%) (**Supplementary Figure 2**), reinforcing their potential relevance as pharmacogenetic markers in AD. Finally, multivariate logistic regression analysis revealed significant associations between generalized eczema phenotype and the combination of FLG rs1328967475 and IL-22 rs1870046714. Of note, a significant association of the combination of FLG rs11204972, FLG rs528128566 and IL6R rs2228145 with response to Dupilumab, in terms of EASI90 achievement, was also revealed (**Supplementary Table 4**).

## 4 Discussion

In this study, we describe the genetic landscape of adult patients with moderate-to-severe AD treated with Dupilumab by integrating clinical, genotypic, and treatment response data to identify potential biomarkers of disease heterogeneity and therapeutic outcomes. In our real-life multicenter study, we simultaneously assessed more than 500 candidate gene variants, with particular attention to epidermal barrier–related genes, namely *FLG* and *KIF3A*, in a cohort of moderate-to-severe AD patients undergoing Dupilumab therapy for one year. Our findings provide evidence supporting the important contribution of barrier gene variants to disease heterogeneity and reinforce their established pathogenic role in AD patients.

Unsupervised clustering of SNP profiles revealed four genetically distinct patient groups, primarily defined by combinatorial patterns of *FLG* and KIF3A genotypes, thus highlighting the contribution of skin barrier genes in shaping AD heterogeneity. Notably, Cluster A (*FLG^+^/KIF3A^+^*), characterized by the concomitant presence of risk alleles in both genes, was strongly associated with a generalized eczema phenotype, potentially reflecting a more severe disease driven by compounded impairment in epidermal barrier integrity.

The pathogenic role of *FLG* and *KIF3A* in AD is well established, given their critical involvement in maintaining skin barrier function (16, 17). Specifically, *FLG* loss-of-function mutations, such as S324X (rs12568784), 2282del4 (rs61816761), R501X (rs558269137), and R2447X (rs138726443), are considered major susceptibility factors in Northern European populations, as they compromise the stratum corneum, increasing trans epidermal permeability to allergens and microbes (18–20). However, the prevalence of these mutations varies markedly by ethnicity, ranging from 8–10% in Northern to 1–4% in Southern Europeans (21–23). In agreement with previous data from Italian cohorts (24), only 2.5% of our patients harbored *FLG*-null alleles.

Importantly, not all *FLG* mutations convey equivalent risk: approximately 40–50% of individuals carrying null alleles never develop eczema (25), suggesting that additional genetic, epigenetic, or environmental modifiers shape the phenotypic expression of *FLG* variants.

Numerous of these loss-of-function variants associating with AD localize within the exon 3 of FLG gene. Most importantly, exon 3 (>12kb) of the *FLG* gene represents a critical exonic region in AD, as it encodes the profilaggrin precursor protein, a key structural and functional component of the keratohyalin granules of epidermal barrier (26–30). Profilaggrin, contains 10 to 12 tandemly repeated filaggrin units and is converted into the intermediate filament–associated protein filaggrin via tightly regulated dephosphorylation and proteolysis. This process generates functional filaggrin monomers that aggregate keratin intermediate filaments, thereby supporting stratum corneum formation and epidermal barrier integrity (31).

In our cohort, several missense non-synonymous SNPs in *FLG* (rs12405241-A1805V, rs12407748-R1891Q, rs1328967475-D3866A, rs7512857-S2020A, rs11584340-P478S, rs58001094-A1167G), differentially distributed across genetic clusters, map to exon 3 of the gene. Among them, the rs1328967475-D3866A variant showed the strongest association with generalized eczema and remained statistically significant after FDR correction. In contrast, the other significant SNPs showed reduced statistical significance after FDR adjustment. This point can represent a limitation of our study, even though it may be related to the size of the study population. This gap may potentially be addressed in analysis including a larger number of patients.

Structural predictions based on I-Mutant V2 and PolyPhen-2 suggested that amino acid substitutions encoded by rs12405241 (A1805V), rs12407748 (R1891Q), and rs7512857 (S2020A) likely reduce FLG protein stability, as predicted *in silico* by I-Mutant-V2 and Poly-Phen 2 tools (data not shown). Likewise, the rs749682384 variant, associated with enhanced response to Dupilumab, was predicted to destabilize *FLG*, pointing to potential pharmacogenetic relevance.

*FLG* variants co-occurred with SNPs in *KIF3A*, a kinesin family gene involved in epithelial barrier maintenance and immune signaling (32, 33). A recent functional study demonstrated that *KIF3A* polymorphisms rs11740584 and rs2299007 introduce novel CpG methylation sites, reducing gene expression, increasing trans epidermal water loss, and promoting AD-like inflammation in *Kif3a*^K14Δ/Δ^ mice. In our cohort, rs11740584 was detected in 25% of patients, predominantly within cluster A, compared to a 50% prevalence in the general population, suggesting an enrichment of this risk variant in clinically severe AD.

Additionally, 11 other *KIF3A* variants, primarily intronic, were found among AD patients, with three SNPs enriched in cluster D (*FLG^-^/KIF3A^+^*). To elucidate the potential functional impact of these variants, we performed expression quantitative trait loci (eQTL) analyses by querying healthy skin tissues in the GTEx portal (GTEx Analysis Release V10). This *in silico* analysis indicated that these variants modulate *KIF3A* and *SEPTIN8* expression in healthy skin (data not shown). SEPTIN8, a cytoskeletal GTPase implicated in epithelial barrier, inflammation and immune regulation (34), may contribute to AD pathophysiology, although its role remains largely unexplored in skin.

We also identified protective variants against generalized eczema within *IL22* (rs1870046714) and *IL5RA* (rs334809). Despite their upstream or intronic locations and lack of eQTL effects, their higher-than-expected frequencies (∼0.8%) in our cohort compared to European reference populations suggest a potential pathogenic or modulatory role in AD. Similarly, *KIF3A* rs1468216, *IL6R* rs2228145, and *RPTN* rs3001978 were all overrepresented in our study population but not clearly linked to disease severity or pruritus, likely reflecting the uniform clinical profile of our cohort (i.e., moderate-to-severe AD requiring systemic treatment).

Notably, the *KIF3A* rs1468216 variant was inversely associated with allergic rhinitis, whereas high IgE levels correlated with the intronic rs543749 SNP in *ADAM33*, a metalloproteinase gene implicated in asthma and airway remodeling (35, 36). This last SNP could reduce the mRNA expression of *ADAM33* itself, as predicted by GTEx analysis. These findings support the hypothesis that certain genetic variants may contribute to shared endotypes across atopic disorders.

One of the most clinically relevant findings of our study was the identification of genetic variants potentially predictive of response to dupilumab. In particular, the missense SNPs *IL6R* rs2228145 (D358A) and *FLG* rs749682384 (A3340V) were strongly associated with a favorable treatment outcome, suggesting their potential role as pharmacogenetic markers. Regarding *FLG*, previous studies reported no significant influence of *FLG* mutations on dupilumab treatment response (37). However, the *FLG* rs749682384 variant identified in our cohort was not investigated in those analyses. In contrast, Clabbers et al. evaluated loss-of-function and missense *FLG* variants in the Dutch AD population, including rs138726443 and rs150597413, which were largely absent in our cohort (< 0.9% for both SNPs).

Conversely, *RPTN* rs3001978 (3′-UTR) and *TSLP* rs2289276 (5′-UTR) variants were associated with suboptimal response, underscoring the need for early identification of likely non-responders to guide alternative therapeutic choices. Notably, rs2289276 SNP in *TSLP*, was identified as eQTL and strongly associated with reduced *TSLP* expression in not exposed and exposed skin tissues.

eQTL analyses reinforced the functional relevance of these associations. For instance, the rs2289276 variant in *TSLP*, which was linked to reduced gene expression, may reflect a blunted inflammatory milieu, insufficient to engage Dupilumab’s mechanism of action. These findings align with our observation that patients with higher baseline EASI scores were more likely to achieve EASI90 at 48 weeks, suggesting that more active Th2-driven inflammation may predict better biologic responsiveness.

## 5 Conclusion

Our findings highlight the potential value of integrating genetic profiling into clinical management and therapeutic decision-making for patients with moderate-to-severe AD. The presence of genetic features, particularly variants in skin barrier genes, was confirmed in our AD patient population, with SNPs in *FLG* and *KIF3A* contributing to the characterization of patients with more aggressive AD phenotypes. This study also identified genetic signatures in *FLG, IL6R, TSLP*, and *RPTN* as potential predictors of Dupilumab response in patients.

As a whole, our data provides a basis for the development of pharmacogenetic tools aimed at personalizing therapy, improving treatment efficacy, and reducing the healthcare burden associated with ineffective treatments. Nonetheless, replication in larger and ethnically different cohorts will be essential to validate these markers and support their translation into routine dermatological practice. Finally, as this study was designed as a candidate SNP study and based on an approach limited by current knowledge of AD genetic susceptibility, broader analyses of additional unbiased genomic regions, will be needed to further expand our findings.

## Data Availability

The datasets analyzed for this study can be found in online repositories as follows: http://ncbi.nlm.nih.gov/bioproject/PRJNA1403633.
