# Supplementary material for: Genetic profiling of patients with atopic dermatitis reveals immune and skin barrier variants associated with generalized eczema and optimal response to Dupilumab therapy
Source: Front Immunol. 2026 Apr 10;17:1788831. doi: 10.3389/fimmu.2026.1788831 (PMC13106602; doi:10.3389/fimmu.2026.1788831)
Supplement: Supplementary file 1 [file DataSheet1.docx]

Supplementary Material

# Supplementary Figures and Tables

## Supplementary Figures


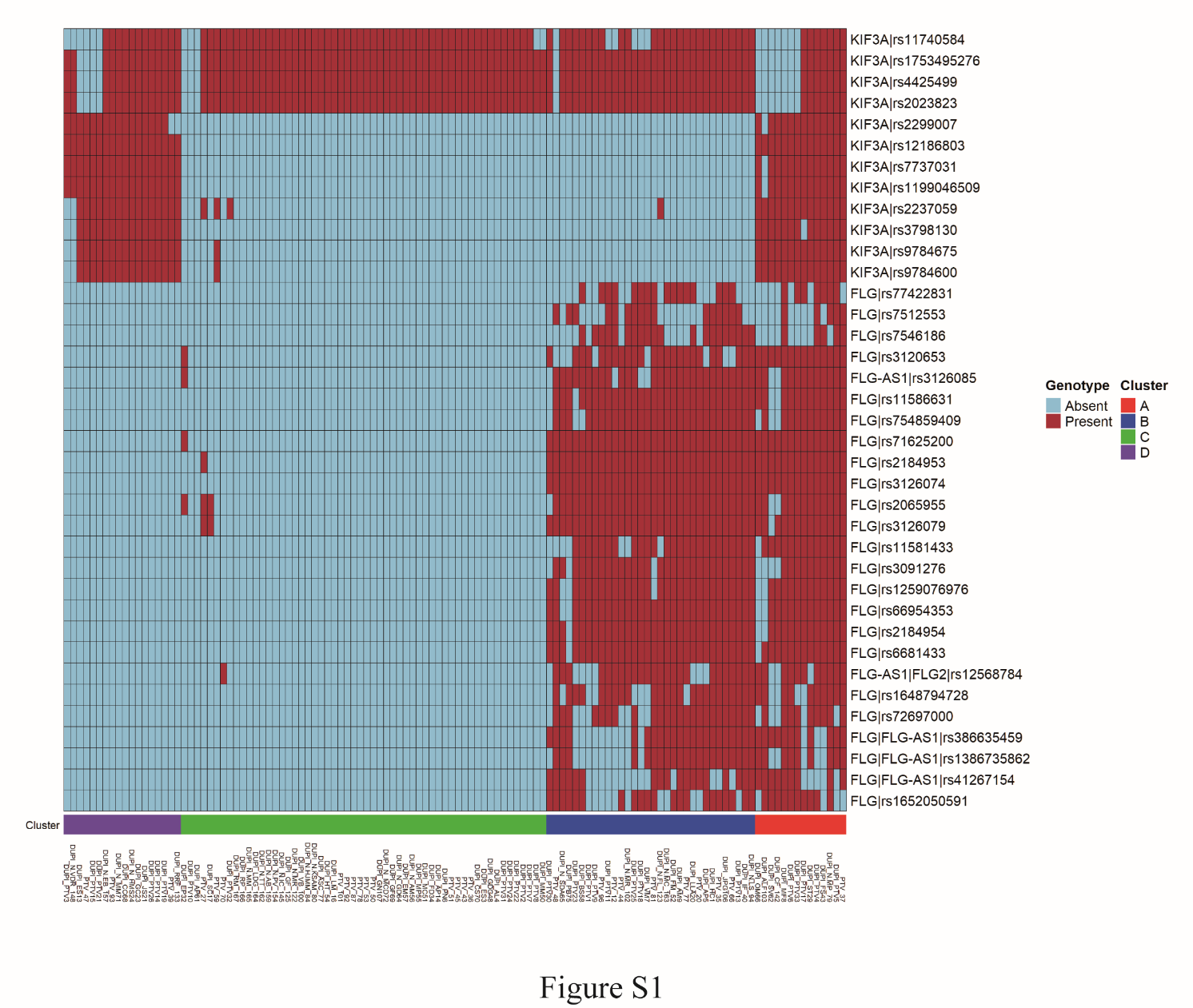


**Supplementary Figure 1.** Heatmap illustrating the distribution of 37 hotspot SNPs located in the *FLG* and *KIF3A* genes within the study cohort. Columns represent individual patients and rows correspond to specific SNP loci. Genotypes are color-coded by the presence or absence of the variant allele. Patients are grouped according to genetic cluster, as indicated by the coloured annotation bar beneath the heatmap.


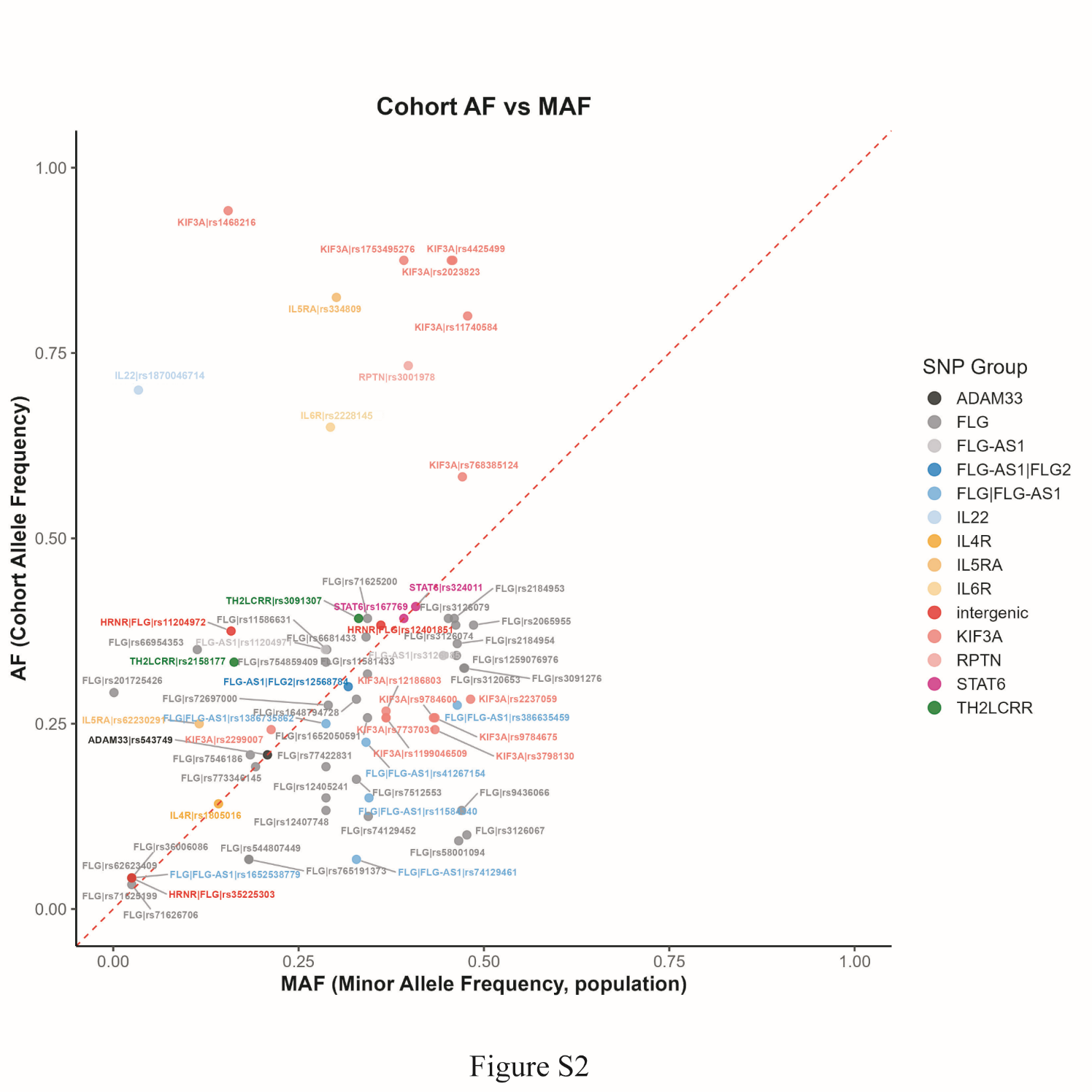


**Supplementary Figure 2.** Scatter plot comparing minor allele frequencies (MAF) in the 1000 Genomes Project global population (*x*-axis) versus the study cohort (*y*-axis) for 76 selected SNPs across multiple genes. Each point corresponds to a variant, coloured by gene of origin. The dashed red diagonal line represents equality (y = x), where cohort and population allele frequencies match. Variant labels indicate gene and specific SNP identity.

## Supplementary Tables

**Table S1. List of SNPs analyzed**

| **Genomic Position** | **Gene** | **REF/ALT** | **dbSNP_ID** | **MAF** | **Heatmap (H,L)** |
| --- | --- | --- | --- | --- | --- |
| chr1:152126467 | RPTN | T/C | rs3001978 | 0.398 | H |
| chr1:152258990 | irFLG | G/C | rs35225303 | 0.025 | - |
| chr1:152259022 | irFLG | T/C | rs12401851 | 0.361 | H |
| chr1:152259078 | irFLG | A/G | rs11204971 | 0.286 | H |
| chr1:152259156 | irFLG | G/A | rs11204972 | 0.159 | H |
| chr1:152274594 | FLG | A/G | rs956511918 | - | - |
| chr1:152274685 | FLG | T/A | rs1933061 | 0.001 | H |
| chr1:152274767 | FLG | G/A | rs114038120 | 0.001 | - |
| chr1:152275183 | FLG | T/C | rs758681801 | - | - |
| chr1:152275272 | FLG | C/T | rs117440780 | 0.005 | - |
| chr1:152275632 | FLG | G/A | rs535144679 | 0.001 | - |
| chr1:152275754 | FLG | C/G | rs778960079 | - | - |
| chr1:152275765 | FLG | T/A | - | - | - |
| chr1:152275765 | FLG | T/G | rs1328967475 | - | H |
| chr1:152275770 | FLG | G/A | - | - | - |
| chr1:152275859 | FLG | T/A | rs766454727 | - | L |
| chr1:152275876 | FLG | C/T | rs145079750 | 0.001 | - |
| chr1:152275883 | FLG | C/A | rs140464988 | 0.001 | - |
| chr1:152275897 | FLG | C/G | rs146684366 | - | - |
| chr1:152276031 | FLG | C/T | rs147295587 | 0 | - |
| chr1:152276062 | FLG | C/T | - | - | - |
| chr1:152276067 | FLG | C/G | rs199804319 | - | - |
| chr1:152276112 | FLG | C/T | rs201725426 | 0.001 | L |
| chr1:152276149 | FLG | C/T | rs77422831 | 0.287 | H |
| chr1:152276156 | FLG | C/T | - | - | - |
| chr1:152276159 | FLG | G/A | rs143135026 | 0.001 | - |
| chr1:152276534 | FLG | G/A | rs772983483 | - | - |
| chr1:152276583 | FLG | C/G | rs12083389 | 0.361 | - |
| chr1:152276657 | FLG | C/G | rs777098884 | - | - |
| chr1:152276660 | FLG | G/A | rs7540123 | 0.207 | - |
| chr1:152276671 | FLG | C/A | rs7518080 | 0.256 | - |
| chr1:152276699 | FLG | A/G | rs12728605 | - | - |
| chr1:152276772 | FLG | C/A | rs72697000 | 0.29 | H |
| chr1:152276848 | FLG | T/C | rs12733038 | 0.012 | - |
| chr1:152276871 | FLG | A/G | rs3126066 | 0.447 | - |
| chr1:152276889 | FLG | A/G | rs3126067 | 0.477 | H |
| chr1:152277055 | FLG | C/G | rs2065955 | 0.486 | H |
| chr1:152277168 | FLG | A/G | rs3091276 | 0.474 | H |
| chr1:152277210 | FLG | C/A | rs78704340 | 0.016 | - |
| chr1:152277226 | FLG | C/G | rs201591837 | 0.001 | - |
| chr1:152277245 | FLG | G/A | rs574781406 | 0 | - |
| chr1:152277248 | FLG | C/T | rs149432670 | 0.001 | - |
| chr1:152277309 | FLG | G/A | rs746608568 | 0.003 | - |
| chr1:152277336 | FLG | A/G | rs373009215 | - | H |
| chr1:152277343 | FLG | G/A | rs749682384 | - | H |
| chr1:152277396 | FLG | T/C | rs6681433 | 0.341 | H |
| chr1:152277405 | FLG | C/T | rs774241945 | - | - |
| chr1:152277554 | FLG | G/A | rs35621145 | 0.025 | - |
| chr1:152277622 | FLG | G/T | rs150597413 | 0.0 | - |
| chr1:152277663 | FLG | C/T | rs140148415 | 0.002 | - |
| chr1:152277717 | FLG | C/A | rs9436066 | 0.47 | H |
| chr1:152277733 | FLG | C/T | rs201718564 | 0.001 | - |
| chr1:152277826 | FLG | A/C | rs2065957 | 0.394 | - |
| chr1:152277848 | FLG | C/T | rs1651886046 | - | - |
| chr1:152277952 | FLG | C/G | rs769504660 | - | - |
| chr1:152277983 | FLG | C/T | rs754064771 | - | - |
| chr1:152278555 | FLG | T/C | rs80221306 | - | - |
| chr1:152278681 | FLG | T/A | - | - | - |
| chr1:152278681 | FLG | T/G | rs1287592834 | - | - |
| chr1:152278689 | FLG | C/A | rs57672167 | 0.396 | - |
| chr1:152278705 | FLG | C/T | rs781033317 | - | - |
| chr1:152278805 | FLG | G/T | rs757231985 | - | - |
| chr1:152278806 | FLG | C/T | rs779224837 | - | - |
| chr1:152278814 | FLG | C/T | rs2184952 | - | - |
| chr1:152278856 | FLG | T/G | rs11582087 | - | L |
| chr1:152278979 | FLG | C/T | rs779385755 | - | - |
| chr1:152279019 | FLG | G/A | rs57670307 | - | - |
| chr1:152279642 | FLG | G/C | rs149024902 | 0.012 | - |
| chr1:152279653 | FLG | T/A | - | - | - |
| chr1:152279653 | FLG | T/G | - | - | - |
| chr1:152279693 | FLG | G/A | rs1557873884 | - | - |
| chr1:152279721 | FLG | C/T | rs12081090 | 0.002 | - |
| chr1:152279729 | FLG | C/T | rs3126072 | 0.475 | - |
| chr1:152279734 | FLG | C/A | rs12081093 | 0.004 | - |
| chr1:152279743 | FLG | C/T | rs148050570 | 0.005 | - |
| chr1:152279816 | FLG | G/C | rs555298118 | 0 | - |
| chr1:152279841 | FLG | G/C | rs3126074 | 0.462 | H |
| chr1:152279920 | FLG | A/G | rs1652050591 | 0.343 | H |
| chr1:152279964 | FLG | C/T | rs71625199 | 0.025 | - |
| chr1:152279975 | FLG | G/C | rs745837741 | 0.004 | - |
| chr1:152280013 | FLG | G/A | rs753498822 | - | - |
| chr1:152280023 | FLG | G/A | rs138726443 | 0.001 | - |
| chr1:152280032 | FLG | T/C | rs71625200 | 0.343 | H |
| chr1:152280110 | FLG | C/T | rs138488969 | 0.005 | - |
| chr1:152280134 | FLG | G/A | rs141651911 | 0.002 | - |
| chr1:152280471 | FLG | C/G | rs78179835 | - | - |
| chr1:152280491 | FLG | C/T | rs200457079 | 0 | - |
| chr1:152280545 | FLG | C/G | rs142969864 | 0 | - |
| chr1:152280573 | FLG | C/T | rs76471961 | 0.049 | - |
| chr1:152280624 | FLG | G/T | rs774174093 | - | - |
| chr1:152280646 | FLG | C/T | rs1652112683 | 0.046 | - |
| chr1:152280685 | FLG | C/A | rs544807449 | 0.183 | L |
| chr1:152280691 | FLG | A/T | rs773346145 | 0.192 | H |
| chr1:152280706 | FLG | C/A | rs1652120260 | - | - |
| chr1:152280736 | FLG | T/C | rs66977240 | - | - |
| chr1:152280759 | FLG | A/G | rs2338554 | 0.367 | - |
| chr1:152280782 | FLG | A/G | rs2184953 | 0.46 | H |
| chr1:152280788 | FLG | T/G | rs66954353 | - | H |
| chr1:152280864 | FLG | A/G | rs2184954 | 0.464 | H |
| chr1:152280900 | FLG | T/G | rs74129452 | 0.344 | H |
| chr1:152280909 | FLG | C/T | rs756319309 | - | - |
| chr1:152280954 | FLG | G/A | rs779803257 | - | - |
| chr1:152281039 | FLG | G/A | rs7522925 | - | - |
| chr1:152281126 | FLG | T/C | rs201446270 | - | - |
| chr1:152281142 | FLG | C/G | rs141120727 | 0.003 | - |
| chr1:152281172 | FLG | T/G | rs74129455 | - | - |
| chr1:152281228 | FLG | C/G | rs7546186 | - | H |
| chr1:152281231 | FLG | G/A | - | - | - |
| chr1:152281290 | FLG | A/G | rs1648794728 | 0.328 | H |
| chr1:152281304 | FLG | A/C | rs7512857 | - | L |
| chr1:152281317 | FLG | G/T | rs748254573 | 0.025 | - |
| chr1:152281423 | FLG | C/T | rs112033499 | - | - |
| chr1:152281446 | FLG | G/C | rs551888992 | 0 | H |
| chr1:152281479 | FLG | G/T | rs3126079 | 0.452 | H |
| chr1:152281523 | FLG | A/C | rs80059102 | - | - |
| chr1:152281534 | FLG | A/T | rs113544881 | 0.314 | - |
| chr1:152281621 | FLG | T/C | rs34806697 | 0.037 | L |
| chr1:152281681 | FLG | C/A | rs1652211221 | - | L |
| chr1:152281690 | FLG | C/T | rs12407748 | 0.287 | H |
| chr1:152281691 | FLG | G/A | rs36006086 | 0.025 | - |
| chr1:152281724 | FLG | G/T | rs149817134 | 0.004 | - |
| chr1:152281745 | FLG | G/T | rs62623409 | 0.025 | - |
| chr1:152281809 | FLG | C/A | rs768262606 | - | - |
| chr1:152281876 | FLG | T/C | rs74129458 | 0.015 | - |
| chr1:152281890 | FLG | C/T | - | - | - |
| chr1:152281947 | FLG | C/T | rs150503906 | 0.011 | - |
| chr1:152281948 | FLG | G/A | rs12405241 | 0.287 | H |
| chr1:152281963 | FLG | C/T | rs566992543 | - | L |
| chr1:152282138 | FLG | G/A | rs747190133 | - | - |
| chr1:152282206 | FLG | G/A | rs774417331 | - | L |
| chr1:152282267 | FLG | G/A | rs12405278 | 0.288 | - |
| chr1:152282311 | FLG | C/T | rs778105843 | 0.297 | - |
| chr1:152282433 | FLG | A/G | rs200129570 | 0 | - |
| chr1:152282616 | FLG | C/T | rs201688513 | 0 | - |
| chr1:152282656 | FLG | C/T | rs750923935 | - | - |
| chr1:152282684 | FLG | G/A | rs151103850 | 0.013 | L |
| chr1:152282794 | FLG | G/A | rs12750081 | 0.025 | - |
| chr1:152282812 | FLG | T/G | rs146433981 | 0.004 | - |
| chr1:152282862 | FLG | C/T | rs1335491195 | - | - |
| chr1:152282909 | FLG | C/T | rs751085109 | 0 | - |
| chr1:152282910 | FLG | G/C | rs71626706 | 0.025 | - |
| chr1:152282917 | FLG | G/T | rs754859409 | 0.287 | H |
| chr1:152282931 | FLG | A/G | - | - | - |
| chr1:152282939 | FLG | T/C | - | - | - |
| chr1:152282952 | FLG | A/C | rs12732920 | 0.025 | - |
| chr1:152282952 | FLG | A/G | rs12732920 | 0.025 | - |
| chr1:152282963 | FLG | C/T | - | - | - |
| chr1:152283053 | FLG | G/A | rs12750571 | 0.005 | - |
| chr1:152283173 | FLG | T/C | rs1348750696 | 0.003 | L |
| chr1:152283175 | FLG | G/C | rs565407815 | 0.001 | L |
| chr1:152283236 | FLG | T/C | rs11581433 | 0.343 | H |
| chr1:152283267 | FLG | T/A | rs11581451 | 0.007 | L |
| chr1:152283283 | FLG | C/T | rs11586631 | 0.288 | H |
| chr1:152283390 | FLG | G/C | rs528128566 | 0 | L |
| chr1:152283396 | FLG | A/T | rs940289052 | - | - |
| chr1:152283466 | FLG | C/T | rs150251062 | 0.002 | - |
| chr1:152283544 | FLG | T/A | - | - | H |
| chr1:152283544 | FLG | T/G | - | - | L |
| chr1:152283549 | FLG | G/A | rs143334387 | 0 | - |
| chr1:152283568 | FLG | T/C | rs11581512 | 0.005 | - |
| chr1:152283625 | FLG | C/A | - | - | - |
| chr1:152283630 | FLG | A/G | - | - | - |
| chr1:152283700 | FLG | C/G | rs202164130 | 0 | - |
| chr1:152283742 | FLG | G/T | rs142660239 | 0.005 | - |
| chr1:152283753 | FLG | C/A | rs141677205 | 0.002 | - |
| chr1:152283808 | FLG | A/G | rs145534539 | - | - |
| chr1:152283846 | FLG | C/T | rs201350571 | - | - |
| chr1:152283862 | FLG | G/C | rs58001094 | 0.466 | H |
| chr1:152283865 | FLG | C/T | rs752322228 | - | - |
| chr1:152283922 | FLG | T/C | rs145475870 | 0.001 | - |
| chr1:152283975 | FLG | A/G | rs1259076976 | 0.463 | H |
| chr1:152284009 | FLG | A/G | - | - | - |
| chr1:152284110 | FLG | C/A | rs770412732 | - | - |
| chr1:152284143 | FLG | A/G | rs771660324 | - | H |
| chr1:152284150 | FLG | G/A | rs1388169688 | - | H |
| chr1:152284177 | FLG | C/T | rs745419566 | - | - |
| chr1:152284269 | FLG | T/G | rs143932553 | 0.001 | - |
| chr1:152284319 | FLG | C/G | rs746364155 | - | H |
| chr1:152284362 | FLG | G/C | rs146262932 | 0 | L |
| chr1:152284424 | FLG | G/C | rs12756586 | 0.034 | - |
| chr1:152284450 | FLG | C/A | rs146104019 | 0.005 | - |
| chr1:152284471 | FLG | C/T | rs756428259 | - | - |
| chr1:152284541 | FLG | G/A | rs138002269 | - | - |
| chr1:152284558 | FLG | C/G | rs150503113 | 0.001 | - |
| chr1:152284576 | FLG | C/A | rs143382793 | - | - |
| chr1:152284595 | FLG | A/G | rs779575711 | - | - |
| chr1:152284649 | FLG | C/T | rs142623038 | 0.003 | - |
| chr1:152284650 | FLG | G/A | rs150580597 | - | - |
| chr1:152284673 | FLG | G/A | rs76586335 | - | - |
| chr1:152284782 | FLG | C/T | rs146300888 | 0.001 | - |
| chr1:152284823 | FLG | A/T | rs74129459 | 0.016 | - |
| chr1:152284854 | FLG | A/G | rs3120653 | 0.473 | H |
| chr1:152284948 | FLG\|FLG-AS1 | G/A | rs72698904 | 0.001 | - |
| chr1:152285021 | FLG\|FLG-AS1 | C/G | rs148739675 | 0.002 | - |
| chr1:152285099 | FLG\|FLG-AS1 | C/T | rs74129461 | 0.328 | L |
| chr1:152285107 | FLG\|FLG-AS1 | C/T | - | - | - |
| chr1:152285119 | FLG\|FLG-AS1 | G/C | rs201522026 | 0.001 | - |
| chr1:152285122 | FLG\|FLG-AS1 | G/A | rs755142336 | - | L |
| chr1:152285181 | FLG\|FLG-AS1 | G/T | rs1652538779 | 0.025 | - |
| chr1:152285238 | FLG\|FLG-AS1 | A/G | rs764214231 | - | - |
| chr1:152285314 | FLG\|FLG-AS1 | C/T | rs111657882 | - | H |
| chr1:152285327 | FLG\|FLG-AS1 | T/G | rs113685999 | 0.003 | - |
| chr1:152285534 | FLG\|FLG-AS1 | C/T | rs74129463 | 0.016 | - |
| chr1:152285555 | FLG\|FLG-AS1 | C/T | rs137995883 | 0.001 | - |
| chr1:152285559 | FLG\|FLG-AS1 | T/C | - | - | - |
| chr1:152285572 | FLG\|FLG-AS1 | C/A | rs766406566 | - | - |
| chr1:152285577 | FLG\|FLG-AS1 | A/G | - | - | - |
| chr1:152285621 | FLG\|FLG-AS1 | T/A | rs145627745 | 0.003 | - |
| chr1:152285654 | FLG\|FLG-AS1 | G/C | rs192402912 | 0 | - |
| chr1:152285759 | FLG\|FLG-AS1 | A/G | rs74129464 | 0.015 | - |
| chr1:152285771 | FLG\|FLG-AS1 | G/A | rs780619446 | - | - |
| chr1:152285878 | FLG\|FLG-AS1 | G/T | rs139321371 | - | - |
| chr1:152285891 | FLG\|FLG-AS1 | C/G | rs761241776 | - | - |
| chr1:152285930 | FLG\|FLG-AS1 | G/A | rs11584340 | 0.345 | H |
| chr1:152285931 | FLG\|FLG-AS1 | C/G | rs527527354 | - | - |
| chr1:152286002 | FLG\|FLG-AS1 | T/C | rs386635459 | 0.464 | H |
| chr1:152286032 | FLG\|FLG-AS1 | C/T | rs1386735862 | 0.287 | H |
| chr1:152286040 | FLG\|FLG-AS1 | C/T | rs76955400 | 0.041 | - |
| chr1:152286097 | FLG\|FLG-AS1 | G/A | - | - | - |
| chr1:152286126 | FLG\|FLG-AS1 | A/G | rs11582620 | 0.086 | L |
| chr1:152286145 | FLG\|FLG-AS1 | G/A | rs189114758 | 0.001 | - |
| chr1:152286204 | FLG\|FLG-AS1 | G/T | rs139754714 | 0.006 | - |
| chr1:152286205 | FLG\|FLG-AS1 | G/C | rs539646707 | 0 | - |
| chr1:152286275 | FLG\|FLG-AS1 | T/A | rs1652634286 | - | H |
| chr1:152286281 | FLG\|FLG-AS1 | C/T | rs150040511 | - | - |
| chr1:152286306 | FLG\|FLG-AS1 | G/A | rs760176246 | - | - |
| chr1:152286308 | FLG\|FLG-AS1 | T/A | rs1652638138 | - | - |
| chr1:152286309 | FLG\|FLG-AS1 | G/C | rs775995278 | - | H |
| chr1:152286367 | FLG\|FLG-AS1 | C/A | rs41267154 | 0.341 | H |
| chr1:152286388 | FLG\|FLG-AS1 | G/A | rs75429799 | 0.045 | - |
| chr1:152286571 | FLG\|FLG-AS1 | T/C | - | - | - |
| chr1:152286798 | FLG\|FLG-AS1 | A/T | - | - | - |
| chr1:152287839 | FLG\|FLG-AS1 | C/A | rs114733570 | 0 | - |
| chr1:152300817 | FLG-AS1 | G/A | rs3126085 | 0.445 | H |
| chr1:152300819 | FLG-AS1 | C/T | rs11590450 | 0.033 | L |
| chr1:152323132 | FLG-AS1\|FLG2 | G/T | rs12568784 | 0.317 | H |
| chr1:152390378 | irFLG-AS1\|FLG2 | A/G | rs6669029 | 0.12 | - |
| chr1:152390452 | irFLG-AS1\|FLG2 | T/C | rs941934 | 0.034 | H |
| chr1:152390515 | irFLG-AS1\|FLG2 | T/C | rs138275012 | 0.001 | - |
| chr1:152770521 | LCE1D | G/A | rs41268492 | 0.337 | - |
| chr1:152770533 | LCE1D | G/A | rs41268494 | - | - |
| chr1:152770589 | LCE1D | A/G | rs11485496 | 0.001 | - |
| chr1:152770613 | LCE1D | T/G | rs41268500 | 0.058 | L |
| chr1:152770647 | LCE1D | C/T | rs765902713 | - | - |
| chr1:152975810 | SPRR3 | G/A | rs527966074 | 0.007 | - |
| chr1:152975829 | SPRR3 | A/G | rs1970328 | 0.475 | - |
| chr1:153520138 | LOC101928034 | C/T | rs41265162 | 0.152 | L |
| chr1:153520203 | LOC101928034 | G/A | rs41265164 | 0.152 | L |
| chr1:154426946 | IL6R | A/T | rs748952018 | - | L |
| chr1:154426947 | IL6R | T/A | rs748952018 | 0.178 | H |
| chr1:154426970 | IL6R | A/C | rs2228145 | 0.293 | H |
| chr2:102926261 | irIL18R1 | C/T | rs62151701 | 0.059 | L |
| chr2:102926320 | irIL18R1 | T/C | rs6721346 | 0.471 | H |
| chr2:102926362 | irIL18R1 | G/A | rs12470864 | 0.302 | H |
| chr2:102926366 | irIL18R1 | G/A | rs12470867 | 0.235 | H |
| chr2:102926430 | irIL18R1 | G/T | - | - | H |
| chr2:102926440 | irIL18R1 | G/C | rs189729232 | 0.002 | - |
| chr2:102971865 | IL18R1 | G/T | rs13015714 | 0.308 | H |
| chr2:102971943 | IL18R1 | C/T | rs539115219 | 0.003 | - |
| chr3:3130221 | IL5RA | A/T | rs334809 | 0.257 | H |
| chr3:3142173 | IL5RA | A/G | rs9831572 | 0.281 | H |
| chr3:3143267 | IL5RA | G/C | rs6771148 | 0.174 | H |
| chr3:3143296 | IL5RA | G/A | rs35418165 | 0.002 | H |
| chr3:3145371 | IL5RA | A/T | rs17881144 | 0.047 | L |
| chr3:3145403 | IL5RA | G/A | rs1703766736 | 0.172 | H |
| chr3:3145424 | IL5RA | T/A | rs78820561 | 0.115 | H |
| chr3:3145527 | IL5RA | A/G | rs62230291 | 0.116 | L |
| chr3:190022484 | CLDN16 | T/G | rs869119629 | 0.175 | L |
| chr3:190022516 | CLDN1 | A/T | rs9290927 | 0.175 | L |
| chr3:190026594 | CLDN1 | G/A | - | - | - |
| chr3:190026654 | CLDN1 | G/T | rs17501010 | 0.143 | L |
| chr3:190026785 | CLDN1 | T/C | rs56328838 | 0.145 | L |
| chr3:190039601 | CLDN1 | G/C | rs893051 | 0.488 | H |
| chr3:190039628 | CLDN1 | T/C | rs79757833 | 0.002 | - |
| chr3:190043739 | CLDN16 | G/A | rs1717050710 | 0.258 | H |
| chr4:100239284 | ADH1B | T/A | rs6413413 | 0.004 | - |
| chr4:100239319 | ADH1B | T/C | rs1229984 | 0.159 | H |
| chr4:154626317 | TLR2 | G/A | rs5743708 | 0.007 | - |
| chr4:154626402 | TLR2 | G/A | rs5743709 | 0.027 | - |
| chr5:110406727 | TSLP | C/T | - | - | - |
| chr5:110406742 | TSLP | C/T | rs3806933 | 0.426 | H |
| chr5:110407507 | TSLP | C/T | rs2289276 | 0.289 | H |
| chr5:110407556 | TSLP | G/C | rs11466738 | 0.014 | - |
| chr5:110407922 | TSLP | A/G | rs572103775 | 0.001 | - |
| chr5:110408002 | TSLP | C/T | rs1898671 | 0.142 | H |
| chr5:110409121 | TSLP | C/T | rs558431039 | 0 | - |
| chr5:110409148 | TSLP | C/G | rs2289278 | 0.135 | L |
| chr5:131984058 | TH2LCRR | A/G | rs2158177 | 0.163 | H |
| chr5:131989060 | TH2LCRR | G/A | rs147753160 | 0.008 | - |
| chr5:131989136 | TH2LCRR | A/G | rs3091307 | 0.331 | H |
| chr5:131995843 | IL13 | T/C | rs1295686 | 0.422 | H |
| chr5:131995964 | IL13 | A/G | rs20541 | 0.27 | H |
| chr5:131996445 | IL13 | A/G | rs1295685 | 0.228 | H |
| chr5:131996500 | IL13 | A/C | rs848 | 0.372 | H |
| chr5:131996597 | IL13 | G/C | rs2069750 | 0.058 | - |
| chr5:132032777 | KIF3A | G/A | rs146726311 | 0.002 | - |
| chr5:132032806 | KIF3A | A/G | rs2023823 | 0.456 | H |
| chr5:132032938 | KIF3A | T/C | rs76311888 | 0.02 | - |
| chr5:132033612 | KIF3A | C/T | rs2237059 | 0.482 | H |
| chr5:132034346 | KIF3A | A/G | rs2023822 | 0.117 | H |
| chr5:132036252 | KIF3A | T/C | rs1468216 | 0.155 | H |
| chr5:132040069 | KIF3A | G/A | rs12186803 | 0.368 | H |
| chr5:132042146 | KIF3A | C/T | rs3798130 | 0.434 | H |
| chr5:132042221 | KIF3A | G/A | - | - | - |
| chr5:132043032 | KIF3A | A/G | rs2299007 | 0.213 | H |
| chr5:132043351 | KIF3A | A/C | rs17691077 | 0.047 | L |
| chr5:132043467 | KIF3A | G/A | rs563313958 | - | - |
| chr5:132046590 | KIF3A | C/T | rs7737031 | 0.368 | H |
| chr5:132046789 | KIF3A | G/C | rs11740584 | 0.478 | H |
| chr5:132046851 | KIF3A | A/G | rs1039637683 | - | - |
| chr5:132049027 | KIF3A | C/T | rs1753495276 | 0.392 | H |
| chr5:132049062 | KIF3A | A/G | rs4425499 | 0.458 | H |
| chr5:132049116 | KIF3A | T/C | rs1199046509 | 0.368 | H |
| chr5:132069706 | KIF3A | G/A | rs9327638 | 0.062 | H |
| chr5:132069739 | KIF3A | A/G | rs9784675 | 0.434 | H |
| chr5:132069800 | KIF3A | C/T | - | - | - |
| chr5:132069847 | KIF3A | G/T | rs9784600 | 0.432 | H |
| chr5:147451572 | SPINK5 | A/G | rs17718511 | 0.052 | L |
| chr5:147466001 | SPINK5 | G/A | rs17860502 | 0.052 | L |
| chr5:147470592 | SPINK5 | G/C | rs72660254 | 0.105 | L |
| chr5:147470679 | SPINK5 | A/T | rs60978485 | 0.052 | L |
| chr5:147479991 | SPINK5 | C/T | rs2303061 | 0.433 | H |
| chr5:147480007 | SPINK5 | A/G | rs2303062 | 0.433 | H |
| chr5:147480013 | SPINK5 | G/A | rs754019195 | - | - |
| chr5:147480027 | SPINK5 | G/A | rs2303063 | 0.431 | H |
| chr5:147480080 | SPINK5 | G/A | rs2303064 | 0.348 | L |
| chr5:147480112 | SPINK5 | T/C | rs2303065 | 0.433 | H |
| chr5:147480868 | SPINK5 | G/A | rs2303066 | 0.433 | H |
| chr5:147480955 | SPINK5 | A/G | rs2303067 | 0.433 | H |
| chr5:147492384 | SPINK5 | T/G | rs72660259 | 0.225 | L |
| chr5:147492461 | SPINK5 | T/C | rs17718737 | 0.054 | L |
| chr5:147494105 | SPINK5 | T/C | rs1422986 | 0.065 | L |
| chr5:147494159 | SPINK5 | A/C | rs1422985 | 0.05 | L |
| chr5:147494160 | SPINK5 | C/T | rs538188629 | 0.002 | - |
| chr5:147499891 | SPINK5 | G/T | rs2303070 | 0.174 | L |
| chr8:6735399 | DEFB1 | C/T | rs11362 | 0.397 | H |
| chr8:6735423 | DEFB1 | C/G | rs1800972 | 0.14 | H |
| chr8:6735431 | DEFB1 | C/T | rs1799946 | 0.446 | H |
| chr8:6735498 | DEFB1 | A/G | - | - | - |
| chr8:6735516 | DEFB1 | C/T | rs56114947 | 0.001 | - |
| chr8:6736496 | DEFB1 | A/T | rs2738181 | 0.28 | H |
| chr8:6736498 | DEFB1 | C/T | rs2738180 | 0.369 | H |
| chr8:6736517 | DEFB1 | T/G | rs2738179 | 0.369 | H |
| chr8:6736520 | DEFB1 | A/C | rs2738178 | 0.37 | H |
| chr8:6736620 | irDEFB1 | C/A | rs5743409 | 0.393 | H |
| chr9:6213193 | irDEFB1 | C/T | rs542013878 | - | - |
| chr9:6213387 | irDEFB1 | G/A | rs928413 | 0.286 | H |
| chr9:27187306 | TEK | C/G | rs2038569 | 0.095 | L |
| chr9:27187332 | TEK | G/T | rs78443822 | 0.066 | L |
| chr9:27187422 | TEK | G/T | rs581724 | 0.416 | H |
| chr9:27187450 | TEK | T/C | rs76779133 | 0.066 | L |
| chr9:120475302 | TLR4 | A/G | rs4986790 | 0.06 | L |
| chr9:120475468 | TLR4 | A/G | rs56070048 | 0.003 | - |
| chr11:65551957 | irOVOL1\|OVOL1-AS1 | A/G | rs479844 | 0.361 | H |
| chr11:65559266 | OVOL1\|OVOL1-AS1 | A/G | rs10791824 | 0.49 | H |
| chr11:76301316 | irOVOL1\|OVOL1-AS1 | C/T | rs7927894 | 0.301 | H |
| chr11:76301348 | irOVOL1\|OVOL1-AS1 | C/T | rs74773769 | 0.004 | - |
| chr11:76301375 | irOVOL1\|OVOL1-AS1 | C/G | rs7927997 | 0.284 | H |
| chr11:76301375 | irOVOL1\|OVOL1-AS1 | C/T | rs7927997 | 0.284 | H |
| chr12:57490100 | NAB2\|STAT6 | T/C | rs324015 | 0.294 | H |
| chr12:57493727 | STAT6 | T/G | rs3024971 | 0.068 | L |
| chr12:57502182 | STAT6 | C/T | rs324011 | 0.275 | H |
| chr12:57503685 | STAT6 | G/T | rs769590814 | - | - |
| chr12:57503775 | STAT6 | C/T | rs167769 | 0.269 | H |
| chr12:68648316 | IL22 | A/G | rs1870046714 | 0.034 | H |
| chr12:68648341 | IL22 | G/A | rs2227481 | 0.153 | L |
| chr12:68648342 | IL22 | G/A | rs2227480 | 0.14 | H |
| chr12:68648356 | IL22 | C/T | rs2227511 | 0.011 | - |
| chr12:68648357 | IL22 | G/A | rs138997944 | 0.002 | - |
| chr16:27373833 | IL4R | C/T | rs6413500 | 0.001 | - |
| chr16:27373872 | IL4R | A/C | rs1805011 | 0.236 | L |
| chr16:27373915 | IL4R | G/T | rs2234898 | 0.236 | L |
| chr16:27373964 | IL4R | T/C | rs1805012 | 0.088 | L |
| chr16:27373966 | IL4R | C/T | rs2234899 | 0.03 | L |
| chr16:27373972 | IL4R | T/C | rs2234900 | 0.263 | L |
| chr16:27373980 | IL4R | C/T | rs1805013 | 0.025 | L |
| chr16:27374400 | IL4R | A/G | rs1801275 | 0.375 | L |
| chr16:27374927 | IL4R | T/G | rs1805016 | 0.124 | L |
| chr16:27375029 | IL4R | T/C | rs1805014 | 0.008 | - |
| chr17:40485229 | STAT3 | T/C | rs1447525119 | 0.081 | - |
| chr17:40485231 | STAT3 | C/T | rs9890635 | 0.081 | - |
| chr17:40485239 | STAT3 | G/T | rs17881320 | 0.036 | L |
| chr17:40485240 | STAT3 | G/C | rs189088246 | 0 | - |
| chr19:7734369 | RETN | C/T | rs3219177 | 0.166 | L |
| chr19:7734409 | RETN | C/T | rs142766550 | 0.001 | - |
| chr19:7734511 | RETN | G/A | rs3745367 | 0.435 | L |
| chr19:10097536 | COL5A3 | G/T | rs8113693 | 0.466 | H |
| chr19:10097593 | COL5A3 | C/T | rs2287808 | 0.124 | H |
| chr19:10097667 | COL5A3 | C/T | rs2287807 | 0.473 | H |
| chr19:10097682 | COL5A3 | G/A | rs2087110837 | - | - |
| chr19:10097740 | COL5A3 | C/T | rs537943098 | 0.0 | - |
| chr20:3649679 | ADAM33 | G/T | rs543749 | 0.189 | L |
| chr20:3649713 | ADAM33 | A/G | rs386811870 | 0.344 | H |
| chr20:3649721 | ADAM33 | T/C | rs2087358573 | 0.342 | H |
| chr20:3649803 | ADAM33 | G/C | rs678881 | 0.191 | H |
| chr20:3650066 | ADAM33 | C/A | rs630712 | 0.16 | H |
| chr20:3650118 | ADAM33 | A/G | rs111935655 | 0.018 | - |
| chr20:3650127 | ADAM33 | G/A | rs2280089 | 0.129 | H |
| chr20:3650205 | ADAM33 | G/A | rs2280090 | 0.144 | H |
| chr20:3650234 | ADAM33 | A/G | rs2280091 | 0.134 | H |
| chr20:3650245 | ADAM33 | G/A | rs145743069 | 0.005 | - |
| chr20:3651742 | ADAM33 | C/G | rs528557 | 0.388 | H |
| chr20:3651765 | ADAM33 | C/T | rs748181775 | 0.048 | L |
| chr20:3651804 | ADAM33 | C/A | rs374116738 | - | - |
| chr20:3655085 | ADAM33 | C/T | rs511898 | 0.464 | H |
| chr20:3655086 | ADAM33 | G/A | rs544032039 | 0 | - |
| chr20:3655219 | ADAM33 | T/C | rs3918392 | 0.067 | L |
| chr20:3655244 | ADAM33 | C/T | rs2087349578 | - | L |
| chr20:44640225 | MMP9 | A/G | rs17576 | 0.456 | H |

*Notes:* Genome position, UCSC Genome Browser hg19 assembly; REF/ALT, reference base/ alteration base; dbSNP_ID, database SNP identification number at NCBI; MAF, minor allele frequency; Heatmap (H,L), SNPs selected for heatmap (High frequency, Low frequency; Chr, chromosome; rs, reference *SNP*ID number; RPTN, Repetin; irFLG, intergenic region of Filaggrin 1; FLG, Filaggrin 1; FLG-AS1, Filaggrin-Antisense1; FLG2, Filaggrin 2; LCE1D, Late Cornified Envelope 1D; SPRR3, Small Proline Rich Protein 3; IL6R, interleukin 6 Receptor; IL18R1, interleukin 18 Receptor1; IL5RA, interleukin 5 ReceptorA; CLDN16, Claudin 16;CLDN1, Claudin 1; ADH1B, alcohol dehydrogenase enzyme 1B; TLR2, toll like receptor 2; TSLP, Thymic Stromal Lymphopoietin; TH2LCRR, T helper type 2 locus control region associated RNA; IL13, interleukin 13; KIF3A, Kinesin Family Member 3A; SPINK5, Serine Peptidase Inhibitor Kazal Type 5; DEFB1, Defensin beta 1; TEK, Receptor Tyrosine Kinase; OVOL1, Ovo Like Transcriptional Repressor 1; OVOL1-AS1, Ovo Like Transcriptional Repressor 1-Antisense 1; NAB2, NGFI-A Binding Protein 1; STAT6, Signal Transducer and Activator of Transcription 6; IL22, interleukin 22; IL4R, interleukin 4 Receptor; STAT3, Signal Transducer and Activator of Transcription; RETN, Resistin; COL5A3, Collagen type 5 alpha 3; ADAM33, metallopeptidase domain 33; MMP9, Matrix Metallopeptidase 9.

**Table S2. Univariable logistic regression for clinical variables and treatment response to Dupilumab**

|  | **Duration of disease** | **IgE levels** | **Bronchial Asthma** | **Conjunctivitis** | **Rhinitis** | **Generalized Eczema** | **EASI _(T0)_** | **NRS Itch** | **NRS Sleep** | **EASI75 _(w48)_** | **EASI90 _(w48)_** |
| --- | --- | --- | --- | --- | --- | --- | --- | --- | --- | --- | --- |
| **Duration of disease** | 1.00 | - | - | - | - | - | - | - | - | - | - |
| **IgE levels** | ns | 1.00 | - | - | - | - | - | - | - | - | - |
| **Bronchial Asthma** | ns | ns | 1.00 | - | - | - | - | - | - | - | - |
| **Conjunctivitis** | ns | ns | OR 4.582 [2.057-10.21]  ***p* < 0.001** | 1.00 | - | - | - | - | - | - | - |
| **Rhinitis** | OR 3.700 [1.739-7.874]  ***p* = 0.001** | ns | OR 4.852 [2.089-11.27]  ***p* < 0.001** | ns | 1.00 | - | - | - | - | - | - |
| **Generalized Eczema** | ns | ns | ns | ns | ns | 1.00 | - | - | - | - | - |
| **EASI _(T0)_** | ns | ns | ns | ns | ns | ns | 1.00 | - | - | - | - |
| **NRS Itch** | ns | ns | ns | ns | ns | ns | ns | 1.00 | - | - | - |
| **NRS Sleep** | ns | OR 4.304 [1.749-10.59]  ***p* = 0.001** | ns | ns | ns | ns | ns | ns | 1.00 | - | - |
| **EASI75 _(w48)_** | ns | ns | ns | ns | ns | ns | ns | ns | ns | 1.00 | - |
| **EASI90 _(w48)_** | ns | ns | ns | ns | ns | ns | OR 6.211 [1.251-30.83]  ***p* = 0.013** | ns | ns | ns | 1.00 |

**Table S3. Presence of Hotspot SNPs by Cluster (%)**

| **Polymorphism** | **Gene** | **dbSNP** | **Cluster A (%)** | **Cluster B (%)** | **Cluster C (%)** | **Cluster D (%)** |
| --- | --- | --- | --- | --- | --- | --- |
| chr5\|132046789\|G\|C | KIF3A | rs11740584 | 50.0 | 81.2 | 91.1 | 66.7 |
| chr5\|132049027\|C\|T | KIF3A | rs1753495276 | 50.0 | 96.9 | 94.6 | 77.8 |
| chr5\|132049062\|A\|G | KIF3A | rs4425499 | 50.0 | 96.9 | 94.6 | 77.8 |
| chr5\|132032806\|A\|G | KIF3A | rs2023823 | 50.0 | 96.9 | 94.6 | 77.8 |
| chr5\|132043032\|A\|G | KIF3A | rs2299007 | 92.9 | 0.0 | 0.0 | 88.9 |
| chr5\|132040069\|G\|A | KIF3A | rs12186803 | **100.0** | 0.0 | 0.0 | **100.0** |
| chr5\|132046590\|C\|T | KIF3A | rs7737031 | 92.9 | 0.0 | 0.0 | **100.0** |
| chr5\|132049116\|T\|C | KIF3A | rs1199046509 | 92.9 | 0.0 | 0.0 | **100.0** |
| chr5\|132033612\|C\|T | KIF3A | rs2237059 | **100.0** | 3.1 | 5.4 | 88.9 |
| chr5\|132042146\|C\|T | KIF3A | rs3798130 | 92.9 | 0.0 | 0.0 | 88.9 |
| chr5\|132069739\|A\|G | KIF3A | rs9784675 | **100.0** | 0.0 | 1.8 | 88.9 |
| chr5\|132069847\|G\|T | KIF3A | rs9784600 | **100.0** | 0.0 | 1.8 | 88.9 |
| chr1\|152276149\|C\|T | FLG | rs77422831 | 50.0 | 50.0 | 0.0 | 0.0 |
| chr1\|152281007\|AA\|GG | FLG | rs7512553 | 35.7 | 50.0 | 0.0 | 0.0 |
| chr1\|152281228\|C\|G | FLG | rs7546186 | 35.7 | 62.5 | 0.0 | 0.0 |
| chr1\|152284854\|A\|G | FLG | rs3120653 | **100.0** | 75.0 | 1.8 | 0.0 |
| chr1\|152300817\|G\|A | FLG-AS1 | rs3126085 | 85.7 | 87.5 | 1.8 | 0.0 |
| chr1\|152283283\|C\|T | FLG | rs11586631 | 85.7 | 93.8 | 0.0 | 0.0 |
| chr1\|152282917\|G\|T | FLG | rs754859409 | 78.6 | 90.6 | 0.0 | 0.0 |
| chr1\|152280032\|T\|C | FLG | rs71625200 | **100.0** | **100.0** | 1.8 | 0.0 |
| chr1\|152280782\|A\|G | FLG | rs2184953 | **100.0** | **100.0** | 1.8 | 0.0 |
| chr1\|152279841\|G\|C | FLG | rs3126074 | **100.0** | **100.0** | 0.0 | 0.0 |
| chr1\|152277055\|C\|G | FLG | rs2065955 | 85.7 | 96.9 | 5.4 | 0.0 |
| chr1\|152281479\|G\|T | FLG | rs3126079 | 92.9 | **100.0** | 3.6 | 0.0 |
| chr1\|152283236\|T\|C | FLG | rs11581433 | 92.9 | 78.1 | 0.0 | 0.0 |
| chr1\|152277168\|A\|G | FLG | rs3091276 | 71.4 | 90.6 | 0.0 | 0.0 |
| chr1\|152283975\|A\|G | FLG | rs1259076976 | 85.7 | 90.6 | 0.0 | 0.0 |
| chr1\|152280788\|T\|G | FLG | rs66954353 | 85.7 | 93.8 | 0.0 | 0.0 |
| chr1\|152280864\|A\|G | FLG | rs2184954 | 85.7 | 96.9 | 0.0 | 0.0 |
| chr1\|152277396\|T\|C | FLG | rs6681433 | 92.9 | 96.9 | 0.0 | 0.0 |
| chr1\|152323132\|G\|T | FLG-AS1\|FLG2 | rs12568784 | 78.6 | 75.0 | 1.8 | 0.0 |
| chr1\|152281290\|A\|G | FLG | rs1648794728 | 71.4 | 75.0 | 0.0 | 0.0 |
| chr1\|152276772\|C\|A | FLG | rs72697000 | 64.3 | 75.0 | 0.0 | 0.0 |
| chr1\|152286002\|T\|C | FLG\|FLG-AS1 | rs386635459 | 78.6 | 68.8 | 0.0 | 0.0 |
| chr1\|152286032\|C\|T | FLG\|FLG-AS1 | rs1386735862 | 64.3 | 65.6 | 0.0 | 0.0 |
| chr1\|152286367\|C\|A | FLG\|FLG-AS1 | rs41267154 | 64.3 | 56.2 | 0.0 | 0.0 |
| chr1\|152279920\|A\|G | FLG | rs1652050591 | 71.4 | 65.6 | 0.0 | 0.0 |

**Table S4. Multivariate Logistic Regression Analysis of clinical variables and**

**treatment response after 48 weeks of Dupilumab**

|  |  | **Univariate analysis** | | | **Multivariate analysis** | | |
| --- | --- | --- | --- | --- | --- | --- | --- |
| **Characteristic** | **Gene \| dbSNP** | **ORs** | **[95% CI]** | ***p-value*** | **ORs** | **[95% CI]** | ***p-value*** |
| Generalized Eczema | FLG\|rs1328967475 | 4.708 | [1.972-11.24] | 0.000 | 3.807 | [1.428-10.15] | 0.008 |
|  | IL5RA\|rs334809 | 0.271 | [0.102-0.717] | 0.009 | ns | ns | ns |
|  | IL22\|rs1870046714 | 0.341 | [0.154-0.757] | 0.009 | 0.358 | [0.149-0.860] | 0.022 |
| EASI90 w48 | irFLG\|rs11204972 | 2.928 | [1.280-6.699] | 0.011 | 3.523 | [1.360-9.126] | 0.010 |
|  | FLG\|rs528128566 | 0.305 | [0.124-0.750] | 0.014 | 0.340 | [0.125-0.921] | 0.034 |
|  | IL6R\|rs2228145 | 3.651 | [1.604-8.308] | 0.002 | 4.725 | [1.856-12.03] | 0.001 |
